# The Burden of Early-Onset Colorectal Cancer and Its Risk Factors from 1990 to 2019: A Systematic Analysis for the Global Burden of Disease Study 2019

**DOI:** 10.3390/cancers14143502

**Published:** 2022-07-19

**Authors:** Wan-Jie Gu, Jun-Peng Pei, Jun Lyu, Naohiko Akimoto, Koichiro Haruki, Shuji Ogino, Chun-Dong Zhang

**Affiliations:** 1 Department of Clinical Research, The First Affiliated Hospital of Jinan University, Guangzhou 510632, China; zlming45@stu2020.jnu.edu.cn (W.-J.G.); lyujun2020@jnu.edu.cn (J.L.); 2 Key Laboratory of Carcinogenesis and Translational Research (Ministry of Education/Beijing), Peking University Cancer Hospital & Institute, Beijing 100142, China; jppei@cmu.edu.cn; 3 Program in MPE Molecular Pathological Epidemiology, Department of Pathology, Brigham and Women’s Hospital and Harvard Medical School, Boston, MA 02115, USA; nakimoto1@bwh.harvard.edu (N.A.); haruki@jikei.ac.jp (K.H.); sogino@bwh.harvard.edu (S.O.); 4 Department of Epidemiology, Harvard T.H. Chan School of Public Health, Boston, MA 02115, USA; 5 Broad Institute of MIT and Harvard, Cambridge, MA 02115, USA; 6 Cancer Immunology and Cancer Epidemiology Programs, Dana-Farber Harvard Cancer Center, Boston, MA 02115, USA; 7 Department of Gastrointestinal Surgery, Graduate School of Medicine, The University of Tokyo, Tokyo 113-8655, Japan

**Keywords:** colorectal carcinoma, global burden of disease, incidence, mortality, young-onset

## Abstract

**Simple Summary:**

In this cross-sectional study, the global incidence, death, and DALY rates of early-onset colorectal cancer (CRC) increased from 1990 to 2019, with large variations in the regional and national levels. A low-milk or low-calcium diet and alcohol use were the leading possible risk factors in 2019. The importance of high body mass index and high fasting plasma glucose increased among both males and females from 1990 to 2019, while the importance of smoking and a low-fiber diet decreased among both sexes, but especially among females. These findings provide policymakers with an accurate quantification of the burden of early-onset CRC, and may help to identify and target high-risk individuals to mitigate the burden of early-onset CRC.

**Abstract:**

Background: The incidence of early-onset colorectal cancer (CRC) diagnosed before age 50 has been increasing over the past decades. Hence, we examined the global, regional, and national burden of early-onset CRC and its risk factors from 1990 to 2019. Methods: Using data from the Global Burden of Disease (GBD) Study 2019, we reported the incidence, deaths, and disability-adjusted life-years (DALYs) attributable to the risk factors of early-onset CRC. All estimates were reported with 95% uncertainty intervals (UIs). Results: The global numbers of early-onset CRC for incidence, deaths, and DALYs in 2019 were 225,736 (95% UI, 207,658 to 246,756), 86,545 (80,162 to 93,431), and 4,259,922 (3,942,849 to 4,590,979), respectively. Despite large variations at the regional and national levels, the global incidence rate, death rate, and DALY rate increased from 1990 to 2019. Diets low in milk, diets low in calcium, and alcohol use were the leading risk factors in 2019. From 1990 to 2019, a high body mass index and high fasting plasma glucose ranked remarkably higher among males and females, while smoking and diets low in fiber ranked lower among both sexes, with a more profound change among females. Conclusions: Despite large variations in regional and national levels, the global incidence rate, death rate, and DALY rate increased during the past three decades. These findings may provide policymakers with an accurate quantification of the burden of early-onset CRC and targeted identification of those most at risk to mitigate the burden of early-onset CRC.

## 1. Introduction

Although the incidence of overall colorectal cancer (CRC) has been declining in the U.S., the incidence of early-onset CRC (EoCRC) diagnosed before 50 years of age has shown a steady increase since the 1980s, resulting in a substantial cancer burden among young adults; however, the reasons for this phenomenon are largely uncertain [1]. Accurate quantifications of the incidence of and death rates from EoCRC remain formidable challenges. Previous studies mainly relied on data from a certain country or region [2,3,4], or data from multiple countries, without information on the disability-adjusted life-years (DALYs) attributable to the risk factors of EoCRC [5]. These limitations thereby hampered the accurate quantification and comparability of the burden of EoCRC across different regions. So far, the incidence or mortality of EoCRC has not been well estimated worldwide, especially at the regional and national levels. The burden of EoCRC may also differ considerably across geographic locations and socioeconomic statuses.

Based on the up-to-date data from the Global Burden of Disease (GBD) Study 2019, [6,7,8] we systematically reported the incidence, deaths, and DALYs of EoCRC and its risk factors at the global, regional, and national levels in relation to year, age, sex, geographic location, and sociodemographic index (SDI) from 1990 to 2019. Although the burden of CRC and its risk factors based on GBD 2019 have been well estimated [9,10,11,12], there is no one study focusing specifically on EoCRC. To our knowledge, this study is the first to investigate the burden of EoCRC and its risk factors based on the latest data from the GBD 2019. Our data may be crucial for policymakers to make better public policy decisions and allocate appropriate resources for cancer prevention, and could also be helpful for the public to reduce exposure to the risk factors of EoCRC.

## 2. Materials and Methods

### 2.1. Data Source

The GBD 2019 provided accessible epidemiological data on 369 diseases and injuries and 87 risk factors from 1990 to 2019, covering 7 super-regions, 21 regions, and over 200 countries and territories. The detailed methodology used for GBD 2019 has been described previously [6,7,8].

### 2.2. Definition of EoCRC

All cancers coded as C18–21, D01.0–D01.2, and D12–D12.9 in the 11th revision of the International Classification of Diseases were considered to be CRCs [13]. We included both colon and rectal carcinomas based on the colorectal continuum model [14]. In this study, EoCRC was defined as CRC diagnosed before 50 years of age. From the Global Health Data Exchange (GHDx) tool (https://ghdx.healthdata.org/gbd-2019, accessed on 15 July 2022), we selected the terms “colon and rectum cancer” as the “cause”, and we selected the terms “incidence”, “deaths”, and “DALYs” as the “measure”.

### 2.3. Estimation of Incidence, Deaths, and DALYs

The estimates of incidence, deaths, and DALYs caused by EoCRC were extracted from the GBD 2019. In this study, the incidence, death, and DALY rates were all reported per 100,000 person-years (per 100,000). All estimates were reported with 95% uncertainty intervals (UIs). Details on the statistical methods are extensively reported (Supplementary Materials) [6].

### 2.4. Sociodemographic Index (SDI)

SDI is a composite indicator of income per capita, average educational attainment, and total fertility rates [8]. The values of SDI range from 0 (worst) to 1 (best), reflecting the degree of socio-development status. We assessed the association between SDI and the incidence rate, death rate, death/incidence ratio, and DALYs attributable to risk factors in EoCRC. Based on the SDI value, geographic locations were classified into high, high-middle, middle, low-middle, and low SDI regions.

### 2.5. Risk Factors

Risk factors that showed evidence of causation with CRC or EoCRC were selected; those included five dietary factors (diet low in milk, diet low in calcium, diet high in red meat, diet high in processed meat, and diet low in fiber) [15,16,17], three behavioral factors (alcohol use, smoking, and low physical activity) [18,19,20], and two metabolic factors (high body mass index and high fasting plasma glucose) [18,21]. Definitions of these risk factors and methods for quantifying the proportions of the burden of EoCRC attributable to these risk factors are described elsewhere (Supplementary Materials) [8]. In brief, the GBD 2019 followed the general framework established for comparative risk assessment to estimate the burden attributable to each risk factor. The DALYs due to EoCRC attributable to each risk factor were estimated by multiplying the total DALYs for EoCRC by the population-attributable fraction for the EoCRC risk–outcome pair for a given age–sex–location–year. The STROBE checklist was applied (www.strobe-statement.org, accessed on 15 July 2022) for this study.

## 3. Results

### 3.1. Incidence, Deaths, and DALYs in 2019

#### 3.1.1. Global Level

**Figure 1:**
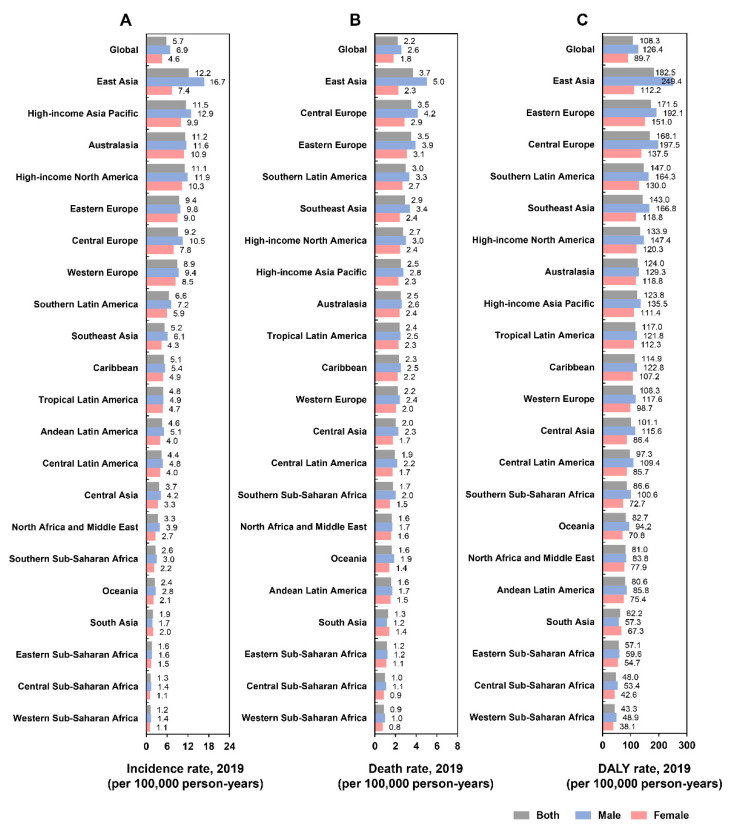
The incidence rate (**A**), death rate (**B**), and DALY rate (**C**) of early-onset CRC worldwide and in 21 GBD regions by sex, 2019. CRC: colorectal cancer; DALY: disability-adjusted life-year; GBD: Global Burden of Disease.

In 2019, the incidence number of EoCRC was 225,736 (95% UI, 207,658 to 246,756), with an incidence rate of 5.7 (5.3 to 6.3) per 100,000 (Figure 1A; Table 1). The death number in 2019 was 86,545 (80,162 to 93,431), with a death rate of 2.2 (2.0 to 2.4) per 100,000 (Figure 1B; Table 1). The DALYs were 4,259,922 (3,942,849 to 4,590,979), with a DALY rate of 108 (100 to 117) per 100,000 (Figure 1C; Table 1).

#### 3.1.2. Regional Level

In 2019, the highest incidence rates per 100,000 of EoCRC were in East Asia (12 [95% UI: 10 to 14]), high-income Asia Pacific (12 [9.9 to 13]), and Australasia (11 [8.8 to 14]),while the lowest were in Western sub-Saharan Africa (1.2 [0.96 to 1.4]), Central sub-Saharan Africa (1.3 [0.91 to 1.7]), and Eastern sub-Saharan Africa (1.6 [1.3 to 1.9]) (Figure 1A; Table 1). The highest death rates per 100,000 were in East Asia (3.7 [3.1 to 4.3]), Central Europe (3.5 [3.0 to 4.0]), and Eastern Europe (3.5 [3.1 to 4.0]), whereas the lowest were in Western sub-Saharan Africa (0.87 [0.69 to 1.1]), Central sub-Saharan Africa (0.97 [0.69 to 1.3]), and Eastern sub-Saharan Africa (1.2 [0.95 to 1.4]) (Figure 1B; Table 1). The highest DALYs per 100,000 were in East Asia (183 [155 to 212]), Eastern Europe (172 [153 to 193]), and Central Europe (168 [144 to 193]), while the lowest were in Western sub-Saharan Africa (43 [35 to 53]), Central sub-Saharan Africa (48 [44 to 65]), and Eastern sub-Saharan Africa (57 [47 to 70]) (Figure 1C; Table 1).

#### 3.1.3. National Level

**Figure 2:**
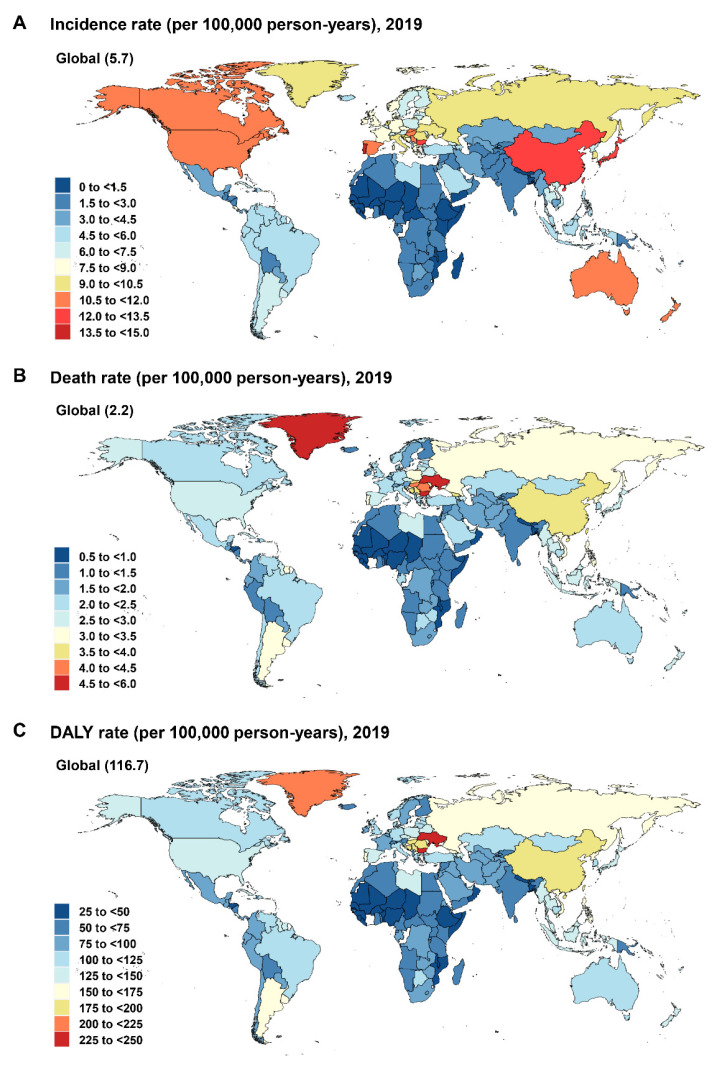
World maps of the incidence rate (**A**), death rate (**B**), and DALY rate (**C**) of early-onset CRC by country and territory, 2019. CRC: colorectal cancer; DALY: disability-adjusted life-year.

In 2019, the highest incidence rates (per 100,000) of EoCRC were in Monaco (15 [95% UI,11 to 20]), Portugal (14 [9.9 to 18]), and Andorra (14 [9.5 to 19]), while the lowest were in Niger (0.73 [0.48 to 1.1]), Somalia (0.93 [0.51 to 1.8]), and Gambia (0.94 [0.61 to 1.4]) (Figure 2A, Table S1). The highest death rates per 100,000 person-years were in Seychelles (5.1 [4.2 to 6.2]), Bulgaria (5.1 [3.9 to 6.7]), and Ukraine (4.7 [3.8 to 5.8]), with the lowest found in Niger (0.55 [0.37 to 0.83]), Gambia (0.68 [0.45 to 0.99]), and Bangladesh (0.70 [0.45 to 1.0]) (Figure 2B, Table S2). The highest DALYs per 100,000 were observed in Seychelles (249 [207 to 299]), Bulgaria (243 [184 to 316]), and Ukraine (227 [186 to 227]), while the lowest were in Niger (28 [18 to 42]), Gambia (34 [23 to 49]), and Bangladesh (34 [22 to 51]) (Figure 2C, Table S3).

The changes (2019 versus 1990) in the incidence rate, death rate, and DALY rate at the global, regional, and national levels are presented in the supplementary results (Table 1; Tables S1–S3; Figures S1A–C and S2A–C).

### 3.2. The Impact of Sex and SDI on Incidence Rate, Death Rate, and DALY Rate

**Figure 3:**
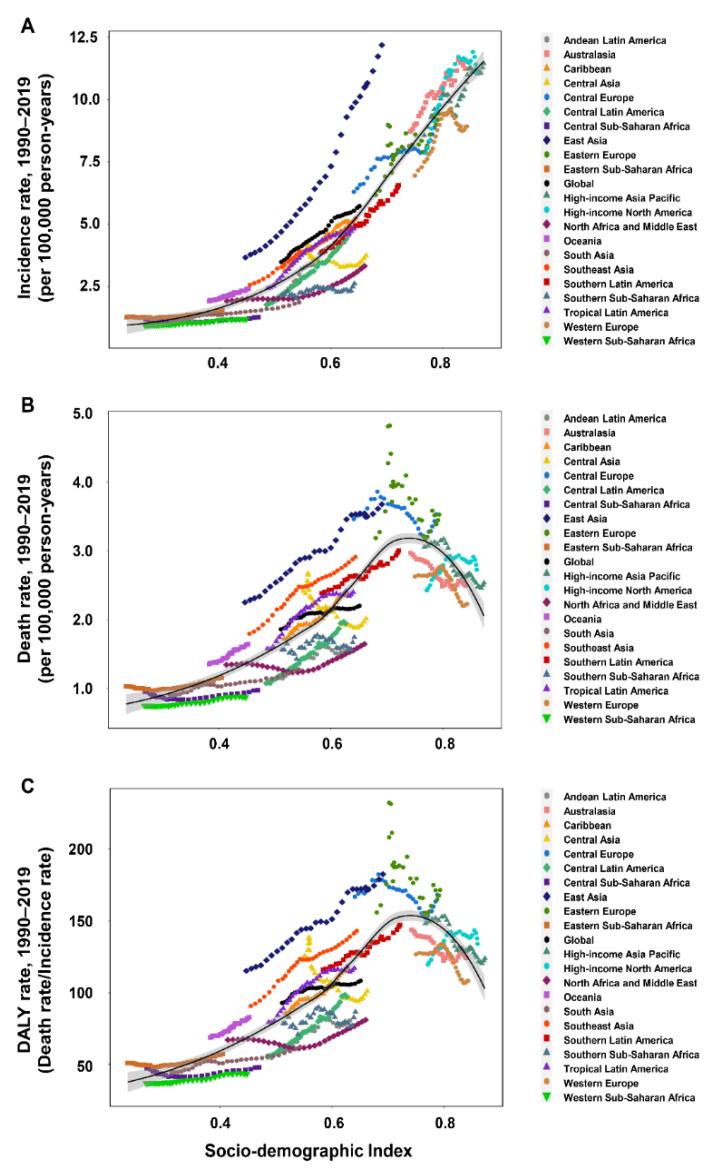
The incidence rate (**A**), death rate (**B**), and DALY rate (**C**) of early-onset CRC worldwide and in 21 GBD regions by SDI, 1990–2019. CRC: colorectal cancer; DALY: disability-adjusted life-year; GBD: Global Burden of Disease; SDI: sociodemographic index.

**Figure 4:**
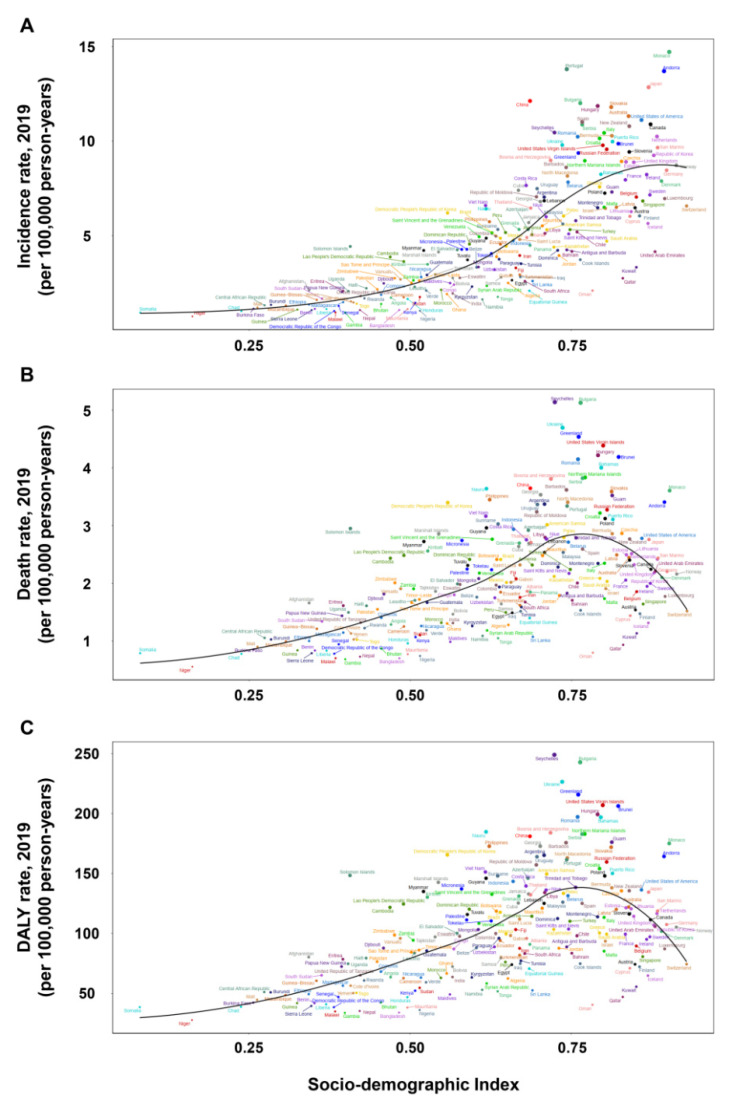
The incidence rate (**A**), death rate (**B**), and DALY rate (**C**) of early-onset CRC at the national level by SDI, 1990–2019. CRC: colorectal cancer; GBD: Global Burden of Disease; SDI: sociodemographic index.

The incidence, death, and DALY rates of EoCRC among males were higher than females in all age groups and regions except South Asia (Figure 1A–C and Figure S3A–C). The incidence rates in most regions and countries showed a rising trend with an increase in the SDI value (Figure 3A and Figure 4A). However, there were some variations. For example, in Western Europe, the incidence rate initially increased remarkably and then decreased with an increase in the SDI value, with a peaked SDI value of 0.815 in 2009. The global incidence rate increased gradually from 1990 to 2019, especially in high-middle, middle, and low-middle SDI regions. In the high SDI region, the incidence rate increased remarkably from 1990 to 2010, followed by a stable trend until 2019 (Figure S4A). For an individual SDI region, the incidence rate showed an upward trend with age (Figure S5A).

The relationship between the death rate and SDI showed obvious regional and national variations (Figure 3B and Figure 4B). In the Caribbean, Central Latin America, East Asia, South Asia, Southern Latin America, and Tropical Latin America, the death rate increased with an increase in the SDI value, whereas in Australasia, it decreased with an increase in the SDI value. In Central Europe, Eastern Europe, Western Europe, and high-income Asia Pacific, the death rate initially increased, then decreased, and finally increased again with an increase in the SDI value. A similar relationship between the DALY rate and SDI was observed at the regional and national levels (Figure 3C and Figure 4C). The global death and DALY rates increased gradually from 1990 to 2019, especially in high-middle and low-middle SDI regions. In the high SDI region, the global death and DALY rates increased remarkably from 1990 to 1995 and then kept a stable trend until 2019 (Figure S4B,C). For individual SDI regions, the death and DALY rates showed an upward trend with an increase in age (Figure S5B,C).

### 3.3. Risk Factors

**Figure 5:**
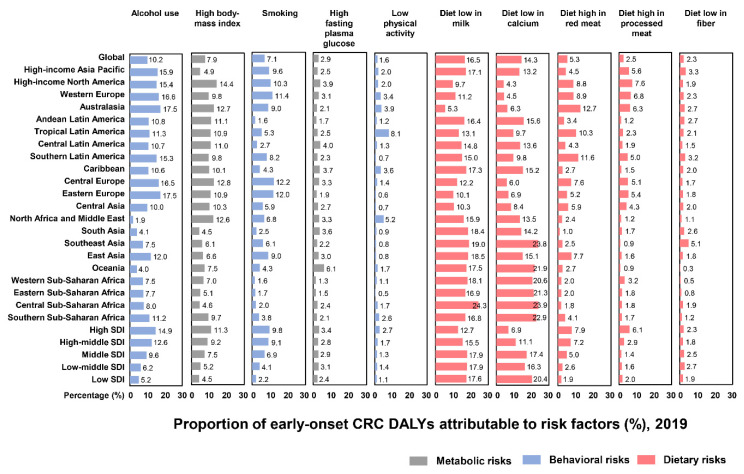
The proportion of DALYs of early-onset CRC to risk factors worldwide and in 21 GBD and 5 SDI regions, 2019. CRC: colorectal cancer; DALYs: disability-adjusted life-years; GBD: Global Burden of Disease; SDI: sociodemographic index.

We included 10 risk factors for DALYs of EoCRC, including five dietary factors, three behavioral factors, and two metabolic factors (Figure 5). Globally, the leading risk factors in 2019 were diets low in milk (17% [95% UI, 11 to 22]), diets low in calcium (17% [11 to 19]), and alcohol use (10% [7.7 to 13]), followed by high body mass index (7.9% [4.3 to 12]), smoking (7.1% [2.6 to 11]), and diets high in red meat (5.3% [1.7 to 9.5]). The remaining risk factors were high fasting plasma glucose (2.9% [0.62 to 6.6]), diets high in processed meat (2.5% [0.86 to 4.0]), diets low in fiber (2.3% [0.94 to 4.2]), and low physical activity (1.6% [0.42 to 3.6]) (Figure 5).

The proportions of DALYs attributable to risk factors of EoCRC differed among regions. The highest percentage of DALYs for alcohol use was in Eastern Europe (18% [14 to 22]), for high body mass index was in high-income North America (14% [8.8 to 20]), for smoking was in Central Europe (12% [4.3 to 19]), for high fasting plasma glucose was in Oceania (6.1% [1.4 to 14]), for low physical activity was in Tropical Latin America (8.1% [1.4 to 15]), for diets low in milk was in Central sub-Saharan Africa (24% [19 to 29]), for diets low in calcium was in Central sub-Saharan Africa (24% [20 to 29%]), for diets low in red meat was in Australasia (13% [6.3 to 19]), for diets low in processed meat was in high-income North America (7.6% [2.9 to 13]), and for diets low in fiber was in Southeast Asia (5.1% [2.4 to 7.7]) (Figure 5).

**Figure 6:**
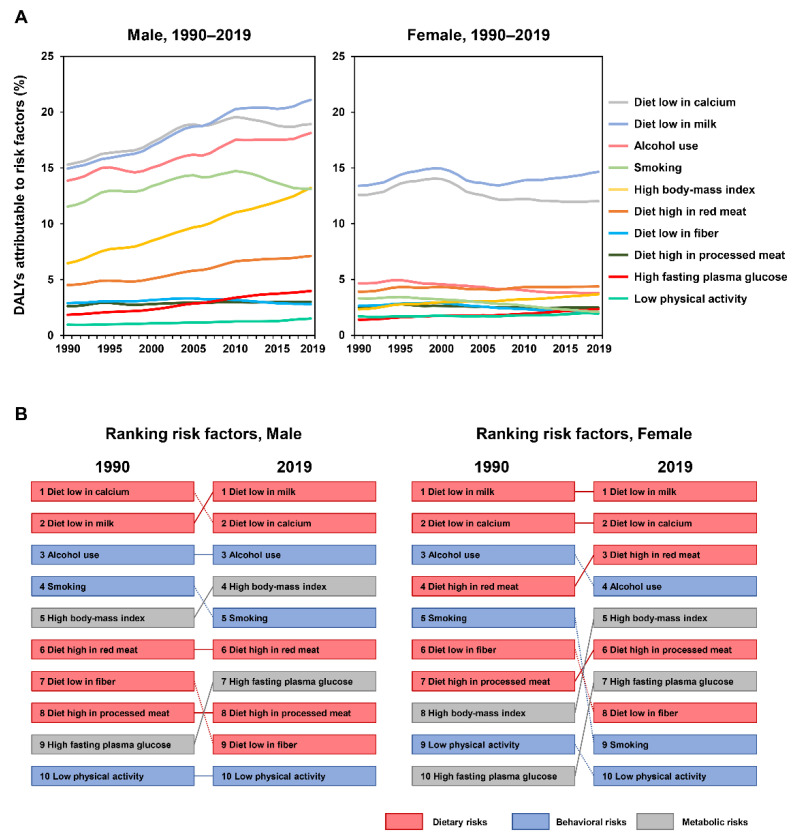
(**A**) The trends of early-onset CRC DALYs compared to risk factors by sex, 1990–2019. (**B**) Comparison of the rankings of early-onset CRC DALYs to risk factors in 1990 and 2019 by sex. CRC: colorectal cancer; DALY: disability-adjusted life-year.

The proportions of DALYs attributable to the risk factors of EoCRC also differed in five levels of SDI regions. For behavioral and metabolic factors, the highest percentage of DALYs was in the high SDI region. For dietary factors, the highest percentage of DALYs for diets low in milk was found in the middle SDI region; for diets low in calcium, the highest percentage was found in the low SDI region; for diets high in red meat and processed meat, the highest percentages were found in the high SDI region; and for diets low in fiber, the highest percentage was found in the low-middle SDI region (Figure 5). The global patterns and the ranking of risk factors among males and females are also presented (Figure 6A,B).

## 4. Discussion

This study systematically analyzed the global, regional, and national burden of EoCRC and its risk factors from 1990 to 2019. Despite the large variations in regional and national levels, the global incidence, death, and DALY rates of EoCRC are increasing. Diets low in milk, diets low in calcium, and alcohol use were the leading risk factors of EoCRC in 2019. From 1990 to 2019, high body mass index and high fasting plasma glucose ranked remarkably higher among males and females, while smoking and diets low in fiber ranked lower among both sexes, with a more profound change among females.

The global incidence rate of CRC increased from 1990 to 2019 by 77.9%, and the global incidence rate increased by 64.3%, with the highest incidence rates in East Asia, high-income Asia Pacific, and Australasia in 2019. Hypothetically, this increase in those regions is potentially associated with socioeconomic development, changes in the Western lifestyle and dietary habits, improvements in health insurance, and the application of a national guideline and screening for CRC [1,22]. The global death rate of CRC increased by 45%, while that of EoCRC increased by 18%. These data suggest differences in the incidence and death rates between EoCRC and later-onset CRC. The reasons for the differences are still unclear, but one potential hypothesis is that the exposures to risk factors for EoCRC are not exactly the same as those for later-onset CRC. Exposures to risk factors of CRC can cause genetic and epigenetic alterations in epithelial cells, and influence the environments of gut microbiota and host immunity [1]. Patients with EoCRC are prone to possess underappreciated clinical symptoms and lack awareness about early screening, resulting in a delayed diagnosis with a more advanced stage [23].

In line with the GBD 2019 Colorectal Cancer Collaborator study [9], the present study found a substantial rise in the EoCRC incidence rate, particularly in the high SDI region, from 1990 to 2019. Globally, diets low in milk (16%), smoking (13%), diets low in calcium (13%), and alcohol use (10%) were the leading risk factors for the whole CRC population in 2019. For the EoCRC population, our study suggested that diets low in milk (17%), diets low in calcium (17%), alcohol use (10%), and high body mass index (8%) were the main contributors. We further found that diets low in milk and diets low in calcium remained the top-ranking factors among both males and females in 2019. The current evidence suggests the importance of a sufficient intake of calcium and milk. High calcium intake demonstrates a protective effect against CRC and EoCRC [20,24,25], possibly due to the role of the extracellular calcium-sensing receptor in anti-tumorigenic effects through down-regulating cellular proliferation and promoting differentiation and apoptosis [26,27]. Diets low in milk have also been associated with a higher risk of CRC [28]. A higher total vitamin D intake was also associated with decreased risk of EoCRC [29]. Taken together, these findings highlight that dietary interventions involving sufficient calcium and milk intake may serve as a potential strategy to alleviate the growing burden of EoCRC.

Notably, two metabolic factors—namely, high body mass index and high fasting plasma glucose—have remarkably ranked higher in their contributions to the burden of EoCRC. A high body mass index, especially obesity, is a strong risk factor for EoCRC. Its increasing prevalence in younger generations substantially contributes to the increase in EoCRC [21]. It was further suggested that obesity was associated with the risk of EoCRC with an odds ratio of 1.4 [30]. A high body mass index during childhood, followed by a pubertal body mass index increase above the median, was associated with an increased risk of colon cancer [31]. Obesity-induced chronic inflammation, gut microbiome reprogramming, and metabolic dysregulation could play important roles in the tumorigenesis of CRC [32,33,34]. Therefore, efforts to control the obesity epidemic, particularly in adolescents and younger adults, may be crucial for preventing EoCRC. High fasting plasma glucose and diabetes were also associated with an increased risk of EoCRC [18,35]. Thus, CRC screening is recommended earlier than the general population for individuals with diabetes [36]. It was revealed that in CRC cells, high glucose levels modulated epithelial-to-mesenchymal transition protein expression and morphology, enhanced cell migration and invasion ability, promoted cell proliferation, and suppressed apoptosis [37,38,39]. Plasma glucose measurements and glycemic control may potentially help to decrease the risk of EoCRC.

Alcohol intake contributes considerably to the burden of EoCRC. It has been suggested that alcohol intake has a positive correlation with EoCRC [18,40]. Regarding EoCRC, the odds ratio was estimated as 1.56 for ≥14 drinks of alcohol per week [16]. Alcohol and its metabolites could exert tumorigenic effects through epigenetic alterations, epithelial barrier dysfunction, and immune-modulatory effects [41], and therefore may be a powerful determinant of EoCRC. Smoking was another risk factor for EoCRC [19], possibly by suppressing T cell-mediated tumor-specific immunity and affecting macrophage functions and polarization to drive colorectal tumorigenesis [42,43]. In our study, smoking ranked lower from 1990 to 2019, especially in females. The global changes may be attributed to the efforts to control smoking in public places worldwide. Besides the above-mentioned risk factors, diets high in processed meat or red meat and diets low in fiber were nongenetic risk factors associated with EoCRC [16,17]. It was currently revealed that an alkylating mutational signature of targeting KRAS p.G12D/p.G13D was associated with red meat consumption and distal CRC location [44]. The results may help with the targeted identification of those most at risk and the mitigation of the rising burden of EoCRC.

Besides the above risk factors from GBD 2019, some other risk factors should also be noted, including sex [45,46], a Westernized diet, antibiotic usage, and alterations in the gut microbiome [47]. The clinicopathological features underlying molecular profiles that act as drivers of EoCRC differ from those of late-onset disease. A substantial proportion of patients with EoCRC may need to receive surgical treatment, which is associated with unfavorable outcomes [48,49]. Moreover, significant risk factors for EoCRC also include a family history of CRC, hyperlipidemia, and inflammatory bowel disease [50,51].

The main strength of this study lies in a comprehensive and up-to-date analysis of the burdens and risk factors of EoCRC in relation to age, sex, location, and the SDI between 1990 and 2019. Due to the inherent deficiencies of GBD 2019, the study still has potential limitations. First, the data from different countries is of different quality, which would inevitably affect the accuracy of estimates. Second, we were unable to determine the burden of EoCRC subtypes by tumor location (proximal colon, distal colon, and rectum) due to a lack of data. Third, some other risk factors of EoCRC (e.g., a Westernized diet, antibiotic usage, and alterations in the gut microbiome) were not evaluated due to a lack of data from GBD 2019, and should be further investigated. Fourth, although we evaluated the risk factors of EoCRC, the lack of data on the thresholds for these risk factors may limit further analyses. Fifth, it has been hypothesized that early-life exposures are risk factors for EoCRC. However, the data on the timing of exposures to risk factors of EoCRC are lacking. Finally, due to the scarce data, we cannot distinguish sporadic EoCRC with a specific type, such as Lynch syndrome.

## 5. Conclusions

In summary, our study showed that the global incidence rate, death rate, and DALY rate of EoCRC increased from 1990 to 2019. There were substantial differences in the incidence rate, death rate, and DALY rate among regions and countries. Risk factors of EoCRC have changed during the past three decades. The leading risk factors in 2019 were diets low in milk, diets low in calcium, and alcohol use. Two metabolic factors, namely high body mass index and high fasting plasma glucose, remarkably ranked higher among males and females from 1990 to 2019, while smoking and diets low in fiber ranked lower among both sexes, with a more profound change among females. Hence, these findings may provide policymakers with an accurate quantification of the burden of EoCRC and allow for the targeted identification of those individuals most at risk to mitigate the burden of EoCRC.

## Figures and Tables

**Table 1:** Incidence, deaths, and DALYs of early-onset colorectal cancer between 1990 and 2019.

Population	Incidence					Deaths					DALYs				
	1990		2019		2019 vs. 1990	1990		2019		2019 vs. 1990	1990		2019		2019 vs. 1990
	Number	Rate	Number	Rate	Rate Change	Number	Rate	Number	Rate	Rate Change	Number	Rate	Number	Rate	Rate Change
	95% UI	per 100,000	95% UI	per 100,000	95% UI	95% UI	per 100,000	95% UI	per 100,000	95% UI	95% UI	per 100,000	95% UI	per 100,000	95% UI
Global	94,707	3.5	22,5736	5.7	64.3	50,437	1.9	86,546	2.2	18.3	2,516,721	92.8	4,259,922	108.3	16.7
	90,421–99,416	3.3–3.7	207,658–246,756	5.3–6.3	49.1 to 81.0	47,475–53,368	1.8–2.0	80,162–93,431	2.0–2.4	7.0 to 29.3	2,368,906–2,663,625	87.3–98.2	3,942,850–4,590,979	100.2–116.7	5.8 to 27.4
Gender															
Male	50,671	3.7	137,138	6.9	86.9	26,990	2.0	51,000	2.6	30.5	134,8146	98.1	2,515,161	126.4	28.9
	47,855–54,342	3.5–4.0	122,715–154,229	6.2–7.8	62.8 to 114.1	25,038–29,445	1.8–2.1	45,983–56,179	2.3–2.8	12.1 to 48.9	1,251,294–1,472,000	91.1–107.1	2,270,969–2,755,869	114.1–138.5	10.7 to 46.6
Female	44,036	3.3	88,598	4.6	38.4	23,447	1.8	35,546	1.8	4.3	1,168,575	87.3	1,744,761	89.7	2.7
	41,091–47,269	3.1–3.5	79,974–97,560	4.1–5.0	22.5 to 55.6	21,543–25,631	1.6–1.9	32,351–38,886	1.7–2.0	−7.7 to 16.5	1,074,665–1,277,269	80.3–95.5	1,587,818–1,910,865	81.6–98.2	−9.0 to 14.7
SDI regions															
High SDI	32,179	7.5	47,490	10.1	35.1	11,544	2.7	12,020	2.6	−4.7	562,074	130.7	589,507	125.4	−4.0
	31,645–32,734	7.4–7.6	43,602–51,743	9.3–11.0	23.8 to 47.8	11,366–11,718	2.6–2.7	11,560–12,509	2.5–2.7	−8.7 to −0.4	552,309–572,092	128.4–133.0	565,599–615,065	120.4–130.9	−8.0 to 0.3
High-middle SDI	27,713	4.6	69,369	9.5	108.0	14,898	2.5	22,183	3.0	23.7	74,2329	122.6	1,094,438	150.2	22.5
	26,289–29,503	4.3–4.9	62,168–77,408	8.5–10.6	83.7 to 135.6	14,014–15,826	2.3–2.6	20,249–24,369	2.8–3.3	10.4 to 37.4	696,956–790,755	115.1–130.6	1,003,070–1,198,241	137.7–164.5	9.7 to 36
Middle SDI	23,378	2.6	68,967	5.5	111.2	15,311	1.7	31,223	2.5	46.0	779,805	86.4	153,8506	122.0	41.2
	21,458–25,471	2.4–2.8	61,737–76,542	4.9–6.1	83.3 to 141.1	13,878–16,640	1.5–1.8	28,191–34,399	2.2–2.7	27.0 to 67.4	707,052–846,544	78.4–93.8	1,390,711–1,693,029	110.3–134.3	23.5 to 61.3
Low-middle SDI	8615	1.6	24,752	2.7	66.4	6433	1.2	15,376	1.6	38.4	321,443	59.5	754,341	80.8	35.9
	7710–9670	1.4–1.8	22,289–27,556	2.4–3.0	41.0 to 92.6	5715–7231	1.1–1.3	13,666–17,137	1.5–1.8	16.1 to 60.7	285,755–361,100	52.9–66.8	672,864–840,215	72.1–90.0	14.5 to 57.3
Low SDI	2777	1.2	7301	1.4	13.1	2226	1.0	5693	1.1	10.1	109,830	47.2	280,703	51.9	10.0
	2284–3327	1.0–1.4	6291–8411	1.2–1.6	−11.5 to 43.9	1836–2671	0.8–1.1	4933–6583	0.9–1.2	−13.4 to 40.2	90,789–131,973	39.0–56.8	242,946–324,266	45.0–60.0	−13.5 to 40.4
GBD regions															
Andean Latin America	351	1.9	1507	4.6	141.3	203	1.1	526	1.6	45.4	10,367	55.7	26,696	80.6	44.8
	311–399	1.7–2.1	1160–1951	3.5–5.9	82.2 to 219.3	181–230	1.0–1.2	411–669	1.2–2.0	10.4 to 90.4	9230–11,732	49.6–63.0	20,869–34,030	63.0–102.7	10.5 to 89.7
Australasia	941	8.7	1520	11.2	28.9	321	3.0	337	2.5	−16.1	15,513	143.7	16,772	124.0	−13.7
	892–993	8.3–9.2	1194–1933	8.8–14.3	0.9 to 63.0	307–335	2.8–3.1	308–370	2.3–2.7	−23.9 to −6.8	14,835–16,199	137.5–150.1	15,269–18,374	112.9–135.9	−22.1 to −4.4
Caribbean	575	3.2	1222	5.1	62.0	314	1.7	562	2.3	36.4	15,629	85.7	27,483	114.9	34.1
	540–611	3.0–3.4	1006–1474	4.2–6.2	32.8 to 94.5	292–339	1.6–1.9	461–681	1.9–2.8	11.9 to 63.5	14,571–16,805	79.9–92.1	22,550–33,298	94.3–139.3	9.9 to 60.7
Central Asia	1297	3.9	1831	3.7	−3.6	806	2.4	983	2.0	−16.6	41,844	125.4	49,357	101.1	−19.4
	1243–1359	3.7–4.1	1637–2062	3.4–4.2	−14.1 to 8.9	773–844	2.3–2.5	878–1114	1.8–2.3	−25.6 to −5.7	40,214–43,859	120.5–131.5	44,065–55,814	90.2–114.3	−28.0 to −8.9
Central Europe	3847	6.3	4850	9.2	45.9	2113	3.5	1861	3.5	2.0	101,778	166.8	88,617	168.1	0.8
	3737–3969	6.1–6.5	4151–5579	7.9–10.6	25.8 to 67.3	2057–2174	3.4–3.6	1598–2134	3.0–4.0	−12.2 to 17.4	99,088–104,830	162.4–171.8	76,070–101,521	144.3–192.6	−13.0 to 15.6
Central Latin America	1452	1.8	5782	4.4	146.5	876	1.1	2564	1.9	81.3	44,894	55.1	128,095	97.3	76.7
	1413–1490	1.7–1.8	4916–6815	3.7–5.2	108.8 to 190.3	853–898	1.0–1.1	2171–3009	1.6–2.3	54.1 to 114.0	43,690–46,041	53.6–56.5	108,759–150,831	82.6–114.5	50.2 to 108.2
Central sub-Saharan Africa	291	1.2	798	1.3	7.5	232	1.0	604	1.0	2.3	11,541	47.3	29,811	48.0	1.4
	215–387	0.9–1.6	566–1082	0.9–1.7	−3.1 to 5.6	177–305	0.7–1.3	428–821	0.7–1.3	−34.6 to 49.1	8753–15,110	35.9–61.9	21,129–40,444	34.0–65.1	−34.9 to 46.6
East Asia	25,348	3.7	90,911	12.2	231.8	15,532	2.2	27,447	3.7	63.5	795,135	115.2	1,362,350	182.5	58.5
	22,185–28,938	3.2–4.2	76,318–106,894	10.2–14.3	16.4 to 31.3	13,405–17,867	1.9–2.6	23,104–32,223	3.1–4.3	30.6 to 102.8	685,952–910,860	99.4–131.9	1,156,497–1,580,832	155.0–211.8	27.9 to 95.4
Eastern Europe	6811	6.2	9239	9.4	52.6	3515	3.2	3459	3.5	10.7	171,933	155.8	168,129	171.5	10.0
	6348–7172	5.8–6.5	8187–10,480	8.3–10.7	35.5 to 72.9	3276–3698	3.0–3.4	3063–3898	3.1–4.0	−1.6 to 24.5	160,508–180,593	145.5–163.7	149,699–189,150	152.7–192.9	−1.9 to 23.6
Eastern sub-Saharan Africa	1067	1.3	3089	1.6	20.8	850	1.0	2298	1.2	12.8	42,134	50.8	113,630	57.1	12.5
	858–1311	1.0–1.6	2526–3770	1.3–1.9	−9.6 to 66.7	678–1043	0.8–1.3	1885–2808	0.9–1.4	−15.7 to 58.8	33,615–51,821	40.5–62.4	92,977–138,693	46.7–69.7	−16.1 to 58.8
High-income Asia Pacific	7953	8.6	9294	11.5	33.8	2837	3.1	2056	2.5	−17.0	137,712	148.3	100,488	123.8	−16.5
	7714–8186	8.3–8.8	8007–10,617	9.9–13.1	15.5 to 53.2	2779–2892	3.0–3.1	1941–2160	2.4–2.7	−21.3 to −12.5	134,351–140,739	144.6–151.5	94,554–105,865	116.5–130.5	−20.9 to −12.1
High-income North America	11,665	7.8	18,499	11.1	41.4	3604	2.4	4545	2.7	12.4	177,662	119.5	223,318	133.9	12.0
	11,348–11,962	7.6–8	15,902–21,547	9.5–12.9	21.3 to 65.6	3517–3688	2.4–2.5	4399–4709	2.6–2.8	7.5 to 18.5	172,981–182,260	116.4–122.6	214,946–232,481	128.9–139.4	7.3 to 18.2
North Africa and Middle East	3126	1.9	11,101	3.3	72.9	2180	1.3	5485	1.6	22.5	108,846	67.0	270,165	81.0	20.8
	2608–3801	1.6–2.3	9616–12,783	2.9–3.8	34.1 to 116.9	1816–2650	1.1–1.6	4716–6362	1.4–1.9	−5.5 to 54.2	90,784–132,718	55.9–81.7	232,244–312,325	69.6–93.6	−6.9 to 51.9
Oceania	61	1.9	165	2.4	25.7	43	1.4	112	1.6	21.6	2174	68.7	5624	82.7	20.4
	49–76	1.5–2.4	126–215	1.9–3.2	−2.7 to 68.9	34–54	1.1–1.7	85–147	1.2–2.2	−6.3 to 64.3	1715–2728	54.2–86.2	4292–7320	63.1–107.6	−6.7 to 61.4
South Asia	6136	1.2	18,253	1.9	61.6	4824	0.9	12,421	1.3	39.9	237,610	44.9	606,012	62.2	38.6
	5397–6949	1.0–1.3	15,671–21,008	1.6–2.2	29.2 to 94.8	4265–5455	0.8–1.0	10,758–14,391	1.1–1.5	12.1 to 68.9	209,984–268,190	39.7–50.7	523,107–698,658	53.7–71.7	11.5 to 66.9
Southeast Asia	6101	2.6	18,976	5.2	103.1	4241	1.8	10,550	2.9	62.4	214,313	90.7	517,741	143.0	57.7
	5195–6824	2.2–2.9	15,675–22,334	4.3–6.2	66.5 to 142.2	3617–4793	1.5–2.0	8800–12,385	2.4–3.4	34.9 to 90.8	181,999–242,558	77.0–102.6	433,010–606,340	119.6–167.5	31.1 to 85.3
Southern Latin America	950	3.9	2233	6.6	69.2	584	2.4	1022	3.0	25.8	28,375	115.8	50,050	147.0	26.9
	903–999	3.7–4.1	1694–2913	5.0–8.6	27.5 to 121.5	558–613	2.3–2.5	936–1116	2.8–3.3	13.7 to 38.9	27,108–29,756	110.7–121.5	45,995–54,610	135.1–160.4	15.3 to 39.7
Southern sub-Saharan Africa	566	2.2	1111	2.6	21.1	406	1.6	738	1.7	12.1	20,452	78.4	36,624	86.6	10.4
	508–631	1.9–2.4	955–1277	2.3–3.0	−1.2 to 45.3	366–452	1.4–1.7	635–852	1.5–2.0	−9.2 to 35.3	18,430–22,833	70.6–87.5	31,528–42,278	99.9–74.5	−10.2 to 33.7
Tropical Latin America	1917	2.4	5755	4.8	97.8	1239	1.6	2857	2.4	51.9	62,339	79.4	139,457	117.0	47.4
	1856–1983	2.4–2.5	5415–6061	4.5–5.1	84.5 to 110.6	1201–1283	1.5–1.6	2698–3003	2.3–2.5	42.1 to 61.5	60,473–64,504	77.0–82.1	131,965–146,425	110.7–122.8	38.4 to 56.3
Western Europe	13,442	6.9	17,021	8.9	28.4	5082	2.6	4247	2.2	−15.2	245,078	126.7	206,454	108.3	−14.6
	13,129–13,761	6.8–7.1	14,619–19,670	7.7–10.3	10.3 to 49.2	4992–5173	2.6–2.7	4053–4426	2.1–2.3	−19.0 to −11.8	240,230–250,088	124.2–129.3	196,144–215,816	102.9–113.2	−18.5 to −10.8
Western sub-Saharan Africa	811	1.0	2577	1.2	25.9	635	0.7	1872	0.9	16.8	31,392	36.9	93,047	43.3	17.4
	642–1009	0.8–1.2	2060–3112	1.0–1.4	−3.7 to 63.7	500–791	0.6–0.9	1494–2316	0.7–1.1	−10.4 to 51.8	24,824–39,038	29.1–45.8	74,217–114,855	34.5–53.4	−9.8 to 52.8

CI: confidence interval; DALYs: disability-adjusted life-years; GBD: global burden of disease; SDI: sociodemographic index; UI: uncertainty interval.

## Data Availability

The data generated and analyzed in this study are available from the Global Health Data Exchange query tool (https://ghdx.healthdata.org/gbd-2019, accessed on 15 July 2022). The data that support the main findings of this study are also available from the corresponding author upon reasonable request.

## Supplementary Materials

The following supporting information can be downloaded at: https://www.mdpi.com/article/10.3390/cancers14143502/s1. Figure S1: The percentage change in the incidence rate (A), death rate (B), and DALY rate (C) of early-onset CRC worldwide and in 21 GBD regions by sex, 2019 versus 1990. CRC: colorectal cancer; DALY: disability-adjusted life-year; GBD: Global Burden of Disease. Error bars indicate 95% uncertainty intervals. Figure S2: World maps of percentage change in the incidence rate (A), death rate (B), and DALY rate (C) of early-onset CRC by country and territory, 2019 versus 1990. CRC: colorectal cancer; DALY: disability-adjusted life-year. Figure S3: Global incidence number and rate (A), death number and rate (B), and DALYs and DALY rate (C) of early-onset CRC by age and sex, 2019. CRC: colorectal cancer; DALYs: disability-adjusted life-years. Figure S4: The trends in incidence rate (A), death rate (B), and DALY rate (C) of early-onset CRC by SDI, 1990–2019. CRC: colorectal cancer; DALY: disability-adjusted life-year; SDI: sociodemographic index. Figure S5: Global incidence number and rate (A), death number and rate (B), and DALYs and DALY rate (C) of early-onset CRC by age and SDI, 2019. CRC: colorectal cancer; DALYs: disability-adjusted life-years; SDI: sociodemographic index. Table S1: Estimates of incidence with its percentage change for early-onset colorectal cancer by geographic locations. Table S2: Estimates of death with its percentage change for early-onset colorectal cancer by geographic locations. Table S3: Estimates of disability-adjusted life-years with its percentage change for early-onset colorectal cancer by geographic locations. References [52,53] are cited in the supplementary materials.
