# Supplementary material for: The Burden of Early-Onset Colorectal Cancer and Its Risk Factors from 1990 to 2019: A Systematic Analysis for the Global Burden of Disease Study 2019
Source: Cancers (Basel). 2022 Jul 19;14(14):3502. doi: 10.3390/cancers14143502 (PMC9323588; doi:10.3390/cancers14143502)
Supplement: Supplementary file 1 [file cancers-14-03502-s001.zip › Supplementary Figures.pdf]

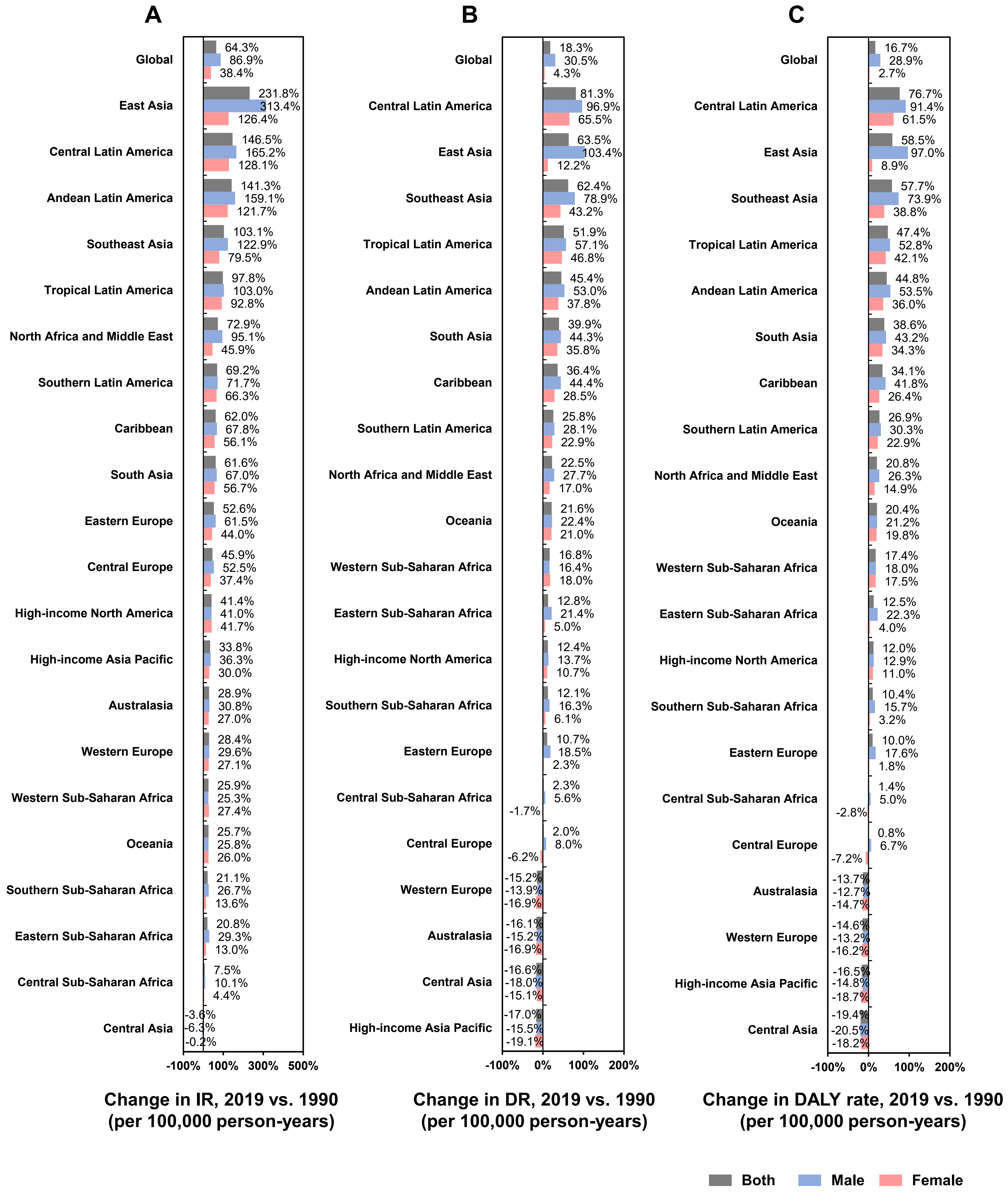
A
Global 64.3% 86.9% 38.4%
East Asia 231.8% 313.4% 126.4%
Central Latin America 146.5% 165.2% 128.1%
Andean Latin America 141.3% 159.1% 121.7%
Southeast Asia 103.1% 122.9% 79.5%
Tropical Latin America 97.8% 103.0% 92.8%
North Africa and Middle East 72.9% 95.1% 45.9%
Southern Latin America 69.2% 71.7% 66.3%
Caribbean 62.0% 67.8% 56.1%
South Asia 61.6% 67.0% 56.7%
Eastern Europe 52.6% 61.5% 44.0%
Central Europe 45.9% 52.5% 37.4%
High-income North America 41.4% 41.0% 41.7%
High-income Asia Pacific 33.8% 36.3% 30.0%
Australasia 28.9% 30.8% 27.0%
Western Europe 28.4% 29.6% 27.1%
Western Sub-Saharan Africa 25.9% 25.3% 27.4%
Oceania 25.7% 25.8% 26.0%
Southern Sub-Saharan Africa 21.1% 26.7% 13.6%
Eastern Sub-Saharan Africa 20.8% 29.3% 13.0%
Central Sub-Saharan Africa 7.5% 10.1% 4.4%
Central Asia -3.6% -6.3% -0.2%
-100% 100% 300% 500%
Change in IR, 2019 vs. 1990
(per 100,000 person-years)
B
Global 18.3% 30.5% 4.3%
Central Latin America 81.3% 96.9% 65.5%
East Asia 63.5% 103.4% 12.2%
Southeast Asia 62.4% 78.9% 43.2%
Tropical Latin America 51.9% 57.1% 46.8%
Andean Latin America 45.4% 53.0% 37.8%
South Asia 39.9% 44.3% 35.8%
Caribbean 36.4% 44.4% 28.5%
Southern Latin America 25.8% 28.1% 22.9%
North Africa and Middle East 22.5% 27.7% 17.0%
Oceania 21.6% 22.4% 21.0%
Western Sub-Saharan Africa 16.8% 16.4% 18.0%
Eastern Sub-Saharan Africa 12.8% 21.4% 5.0%
High-income North America 12.4% 13.7% 10.7%
Southern Sub-Saharan Africa 12.1% 16.3% 6.1%
Eastern Europe 10.7% 18.5% 2.3%
Central Sub-Saharan Africa 2.3% 5.6% -1.7%
Central Europe 2.0% 8.0% -6.2%
Western Europe -15.2% -13.9% -16.9%
Australasia -16.1% -15.2% -16.9%
Central Asia -16.6% -18.0% -15.1%
High-income Asia Pacific -17.0% -15.5% -19.1%
-100% 0% 100% 200%
Change in DR, 2019 vs. 1990
(per 100,000 person-years)
C
Global 16.7% 28.9% 2.7%
Central Latin America 76.7% 91.4% 61.5%
East Asia 58.5% 97.0% 8.9%
Southeast Asia 57.7% 73.9% 38.8%
Tropical Latin America 47.4% 52.8% 42.1%
Andean Latin America 44.8% 53.5% 36.0%
South Asia 38.6% 43.2% 34.3%
Caribbean 34.1% 41.8% 26.4%
Southern Latin America 26.9% 30.3% 22.9%
North Africa and Middle East 20.8% 26.3% 14.9%
Oceania 20.4% 21.2% 19.8%
Western Sub-Saharan Africa 17.4% 18.0% 17.5%
Eastern Sub-Saharan Africa 12.5% 22.3% 4.0%
High-income North America 12.0% 12.9% 11.0%
Southern Sub-Saharan Africa 10.4% 15.7% 3.2%
Eastern Europe 10.0% 17.6% 1.8%
Central Sub-Saharan Africa 1.4% 5.0% -2.8%
Central Europe 0.8% 6.7% -7.2%
Australasia -13.7% -12.7% -14.7%
Western Europe -14.6% -13.2% -16.2%
High-income Asia Pacific -16.5% -14.8% -18.7%
Central Asia -19.4% -20.5% -18.2%
-100% 0% 100% 200%
Change in DALY rate, 2019 vs. 1990
(per 100,000 person-years)
Both
Male
Female

Figure S1

A **Percentage change in incidence rate, 2019 vs. 1990**

Global (64.3%)

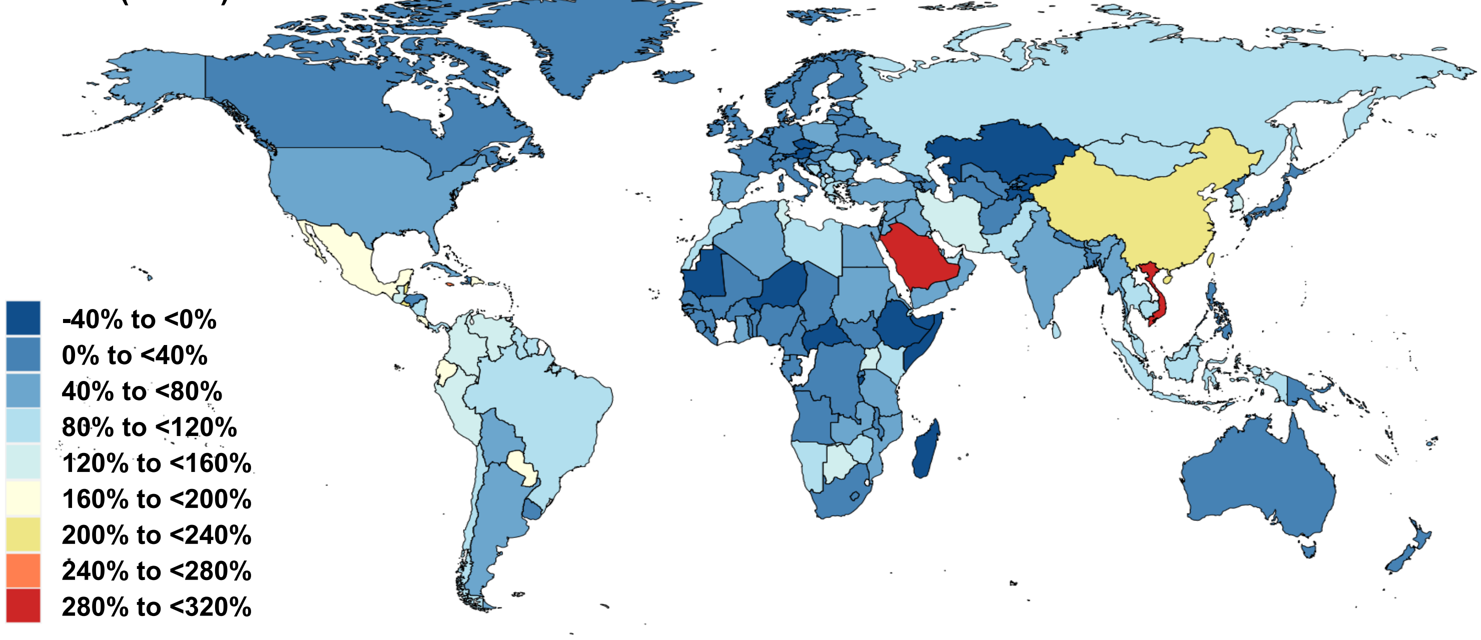

B **Percentage change in death rate, 2019 vs. 1990**

Global (18.3%)

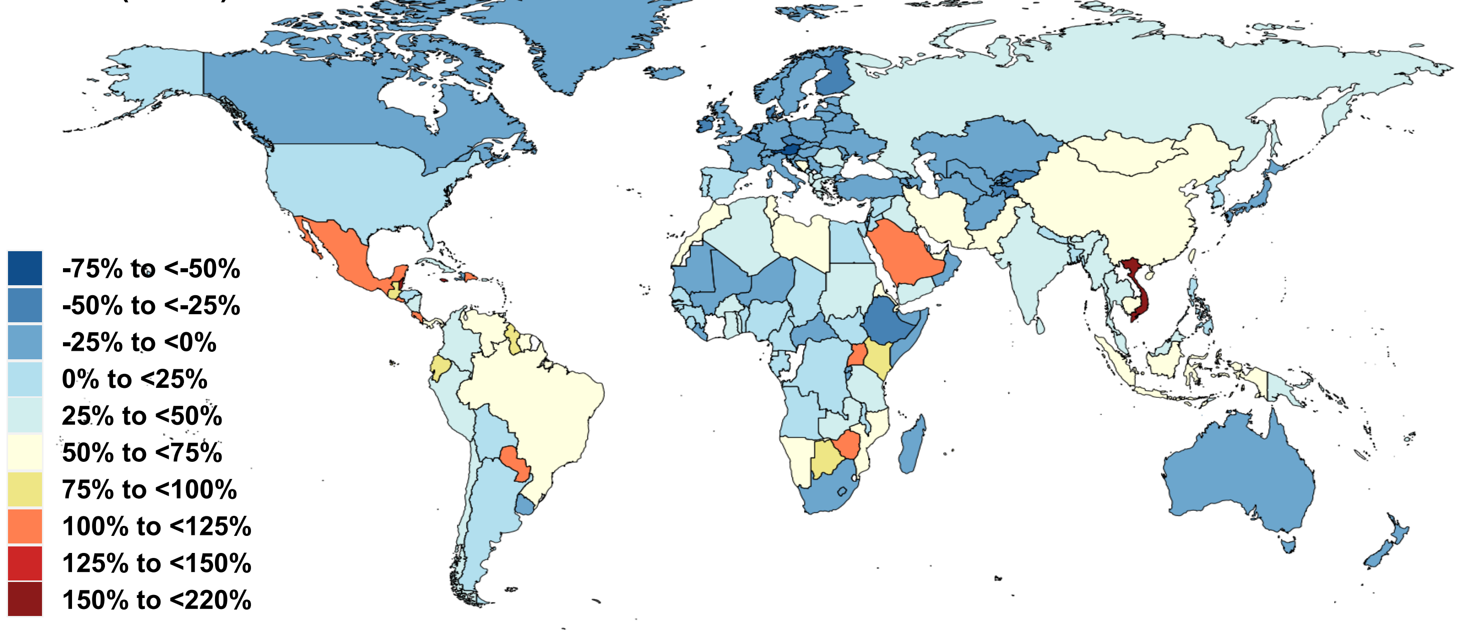

C **Percentage change in DALY rate, 2019 vs. 1990**

Global (16.7%)

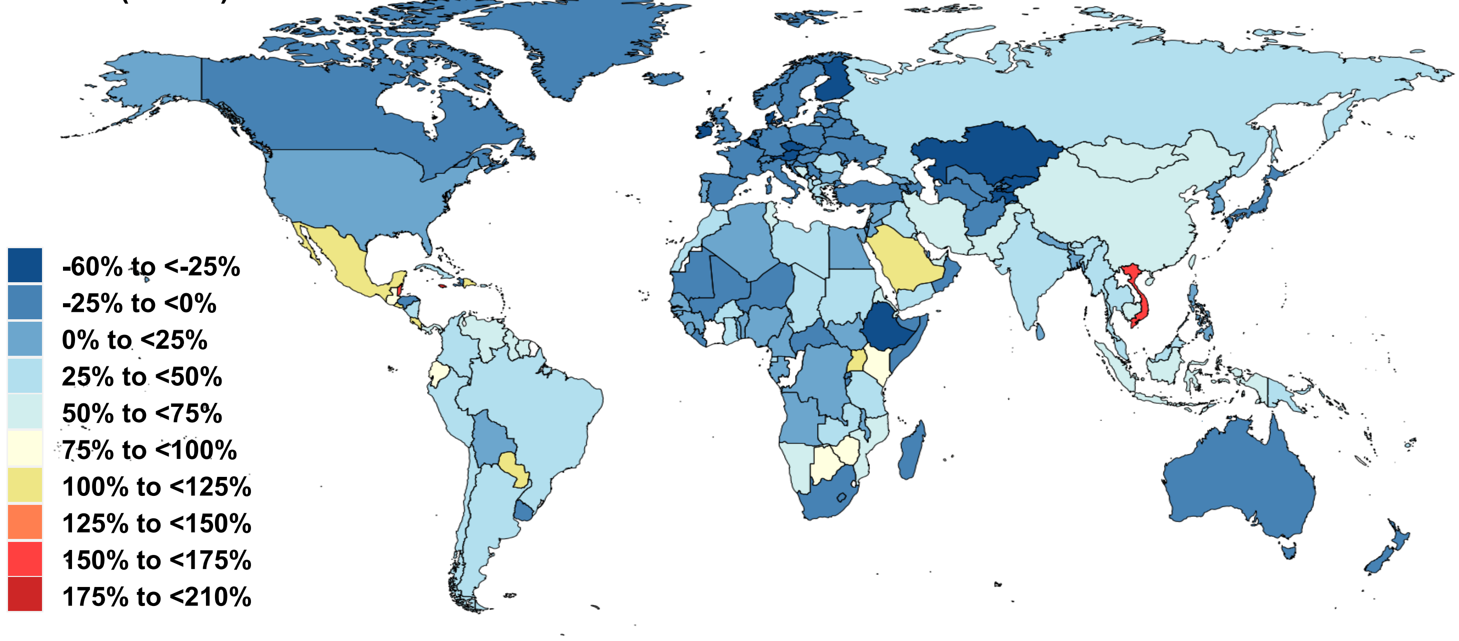

Figure S2

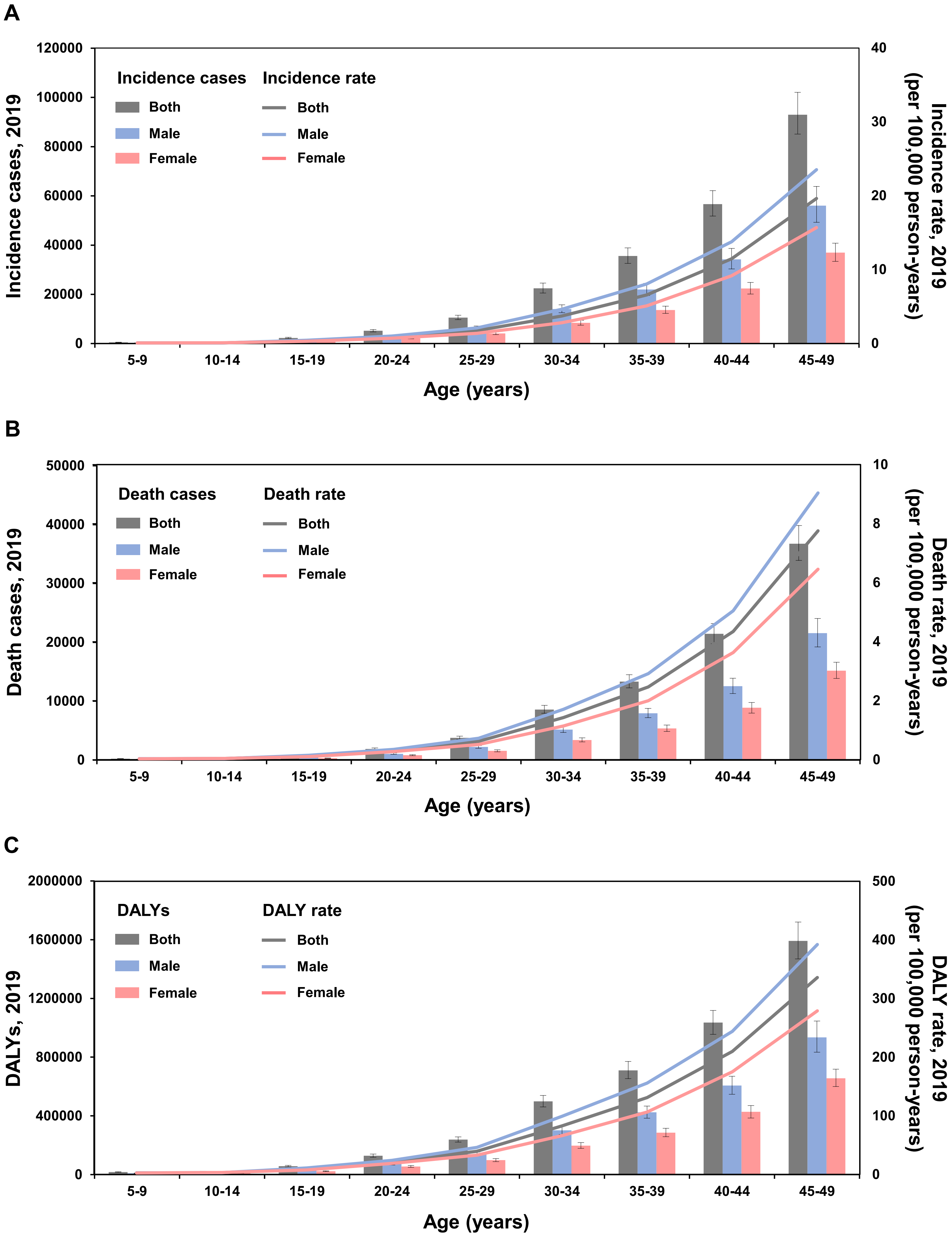
A
Incidence cases
Incidence rate
Both
Male
Female
Both
Male
Female
Incidence cases, 2019
Incidence rate, 2019 (per 100,000 person-years)
120000
100000
80000
60000
40000
20000
0
40
30
20
10
0
5-9
10-14
15-19
20-24
25-29
30-34
35-39
40-44
45-49
Age (years)
B
Death cases
Death rate
Both
Male
Female
Both
Male
Female
Death cases, 2019
Death rate, 2019 (per 100,000 person-years)
50000
40000
30000
20000
10000
0
10
8
6
4
2
0
5-9
10-14
15-19
20-24
25-29
30-34
35-39
40-44
45-49
Age (years)
C
DALYs
DALY rate
Both
Male
Female
Both
Male
Female
DALYs, 2019
DALY rate, 2019 (per 100,000 person-years)
2000000
1600000
1200000
800000
400000
0
500
400
300
200
100
0
5-9
10-14
15-19
20-24
25-29
30-34
35-39
40-44
45-49
Age (years)

Figure S3

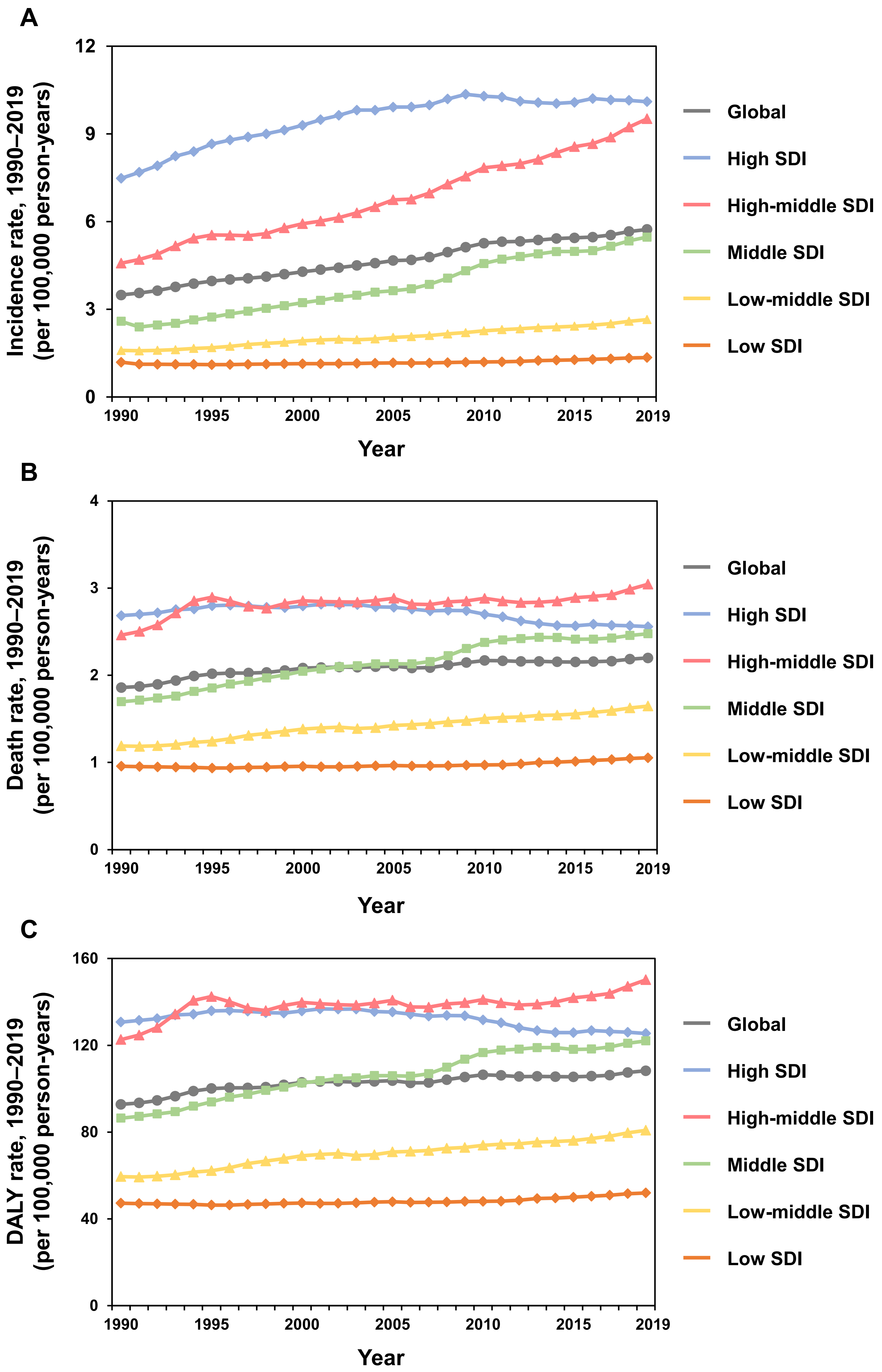
A
Incidence rate, 1990–2019
(per 100,000 person-years)
12
9
6
3
0
1990
1995
2000
2005
2010
2015
2019
Year
Global
High SDI
High-middle SDI
Middle SDI
Low-middle SDI
Low SDI
B
Death rate, 1990–2019
(per 100,000 person-years)
4
3
2
1
0
1990
1995
2000
2005
2010
2015
2019
Year
Global
High SDI
High-middle SDI
Middle SDI
Low-middle SDI
Low SDI
C
DALY rate, 1990–2019
(per 100,000 person-years)
160
120
80
40
0
1990
1995
2000
2005
2010
2015
2019
Year
Global
High SDI
High-middle SDI
Middle SDI
Low-middle SDI
Low SDI

Figure S4

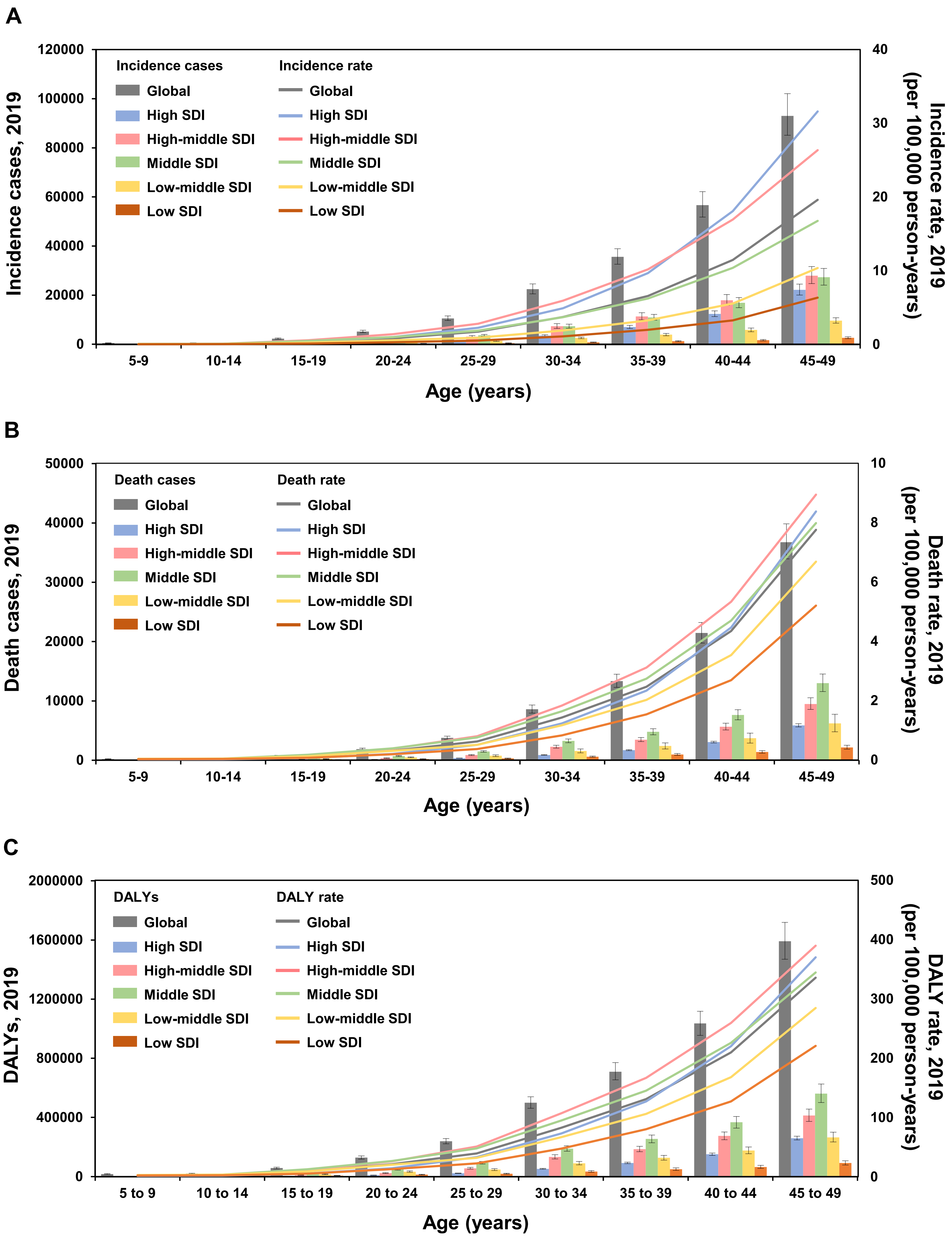
A
Incidence cases
Incidence rate
Global
High SDI
High-middle SDI
Middle SDI
Low-middle SDI
Low SDI
Incidence cases, 2019
Incidence rate, 2019 (per 100,000 person-years)
0
20000
40000
60000
80000
100000
120000
0
10
20
30
40
5-9
10-14
15-19
20-24
25-29
30-34
35-39
40-44
45-49
Age (years)
B
Death cases
Death rate
Global
High SDI
High-middle SDI
Middle SDI
Low-middle SDI
Low SDI
Death cases, 2019
Death rate, 2019 (per 100,000 person-years)
0
10000
20000
30000
40000
50000
0
2
4
6
8
10
5-9
10-14
15-19
20-24
25-29
30-34
35-39
40-44
45-49
Age (years)
C
DALYs
DALY rate
Global
High SDI
High-middle SDI
Middle SDI
Low-middle SDI
Low SDI
DALYs, 2019
DALY rate, 2019 (per 100,000 person-years)
0
400000
800000
1200000
1600000
2000000
0
100
200
300
400
500
5 to 9
10 to 14
15 to 19
20 to 24
25 to 29
30 to 34
35 to 39
40 to 44
45 to 49
Age (years)

Figure S5
