# Supplementary material for: The Burden of Early-Onset Colorectal Cancer and Its Risk Factors from 1990 to 2019: A Systematic Analysis for the Global Burden of Disease Study 2019
Source: Cancers (Basel). 2022 Jul 19;14(14):3502. doi: 10.3390/cancers14143502 (PMC9323588; doi:10.3390/cancers14143502)
Supplement: Supplementary file 1 [file cancers-14-03502-s001.zip › Supplementary methods.pdf]

Supplementary methods

Estimation of incidence, death, and disability-adjusted life years

In GBD 2019, modelling cause-specific mortality was the first step in estimating the burden of cancer. Data were derived from vital registration systems, vital registration samples, verbal autopsy, and cancer registry data. [1,2] Vital registration systems recorded vital event data for all residents by governments, including causes of death. Vital registration samples recorded vital event data in nationally representative cluster samples to estimate birth rates, deaths rates, and causes of death for the total population in countries where vital registration systems were not available. Verbal autopsy involved the collection of information by trained interviewers relating to the signs, symptoms, and demographic characteristics of a recently deceased person from an individual familiar with the deceased, to determine the causes of death and cause-specific mortality fractions in populations without complete vital registration. Cancer registries include data on all cancer patients in a defined population, typically from a particular location.

Of these, only cancer registries include both mortality and incidence data, whereas vital registration and verbal autopsy only provide mortality data. [1,2] Because mortality data from vital registration and verbal autopsy were scarce for some locations and time points, mortality data were also estimated from the cancer registry incidence data with separately modelled mortality-to-incidence ratios (MIRs). As the next step, both observed mortality (from vital registration and verbal autopsy) and estimated mortality (computed from the MIRs and incidence data) data were then used as inputs for a Cause of Death Ensemble model (CODEm), which predicts single-cause mortality based on the available data and covariates with a causal relationship. [1,2]

Furthermore, CoDCorrect was applied to measure single-cause to all-cause mortality estimates to ensure that all single-cause mortality estimates matched the separately modelled all-cause mortality estimates. [3] The incidence of each cancer was calculated by dividing the cause-specific mortality estimates by the MIRs. The final mortality estimates were divided by the estimated MIRs to compute the incidence. Disability-adjusted life years (DALYs) were calculated by summing years lived with disability (YLDs) and years of life lost (YLLs). YLDs were estimated by dividing 10-year cancer prevalence into four sequelae and multiplying the prevalences of these sequelae by the corresponding disability weights: diagnosis and primary therapy, controlled phase, metastatic

phase, and terminal phase. YLLs were estimated by multiplying the estimated number of deaths by age with a standard life expectancy at that age. [1]

Possible risk factors

The GBD 2019 followed the general framework established for comparative risk assessment (CRA) to estimate the burden attributable to each risk factor. CRA can be divided into six key steps: determining the inclusion of risk-outcome pairs; estimating relative risk as a function of exposure for each risk-outcome pair; estimating the distribution of exposure for each risk by age-sex-location-year; determining the level of exposure with minimum risk called the theoretical minimum risk exposure level (TMREL); estimating the population attributable fraction (PAF) and attributable burden; and estimating the PAF and attributable burden for combinations of risk factors. Details of each step have been described elsewhere. [4] Four metrics of burden attributable to a specific risk factor were assessed: deaths, YLDs, YLLs, and DALYs. For instance, to estimate the DALYs due to early-onset CRC attributable to a specific risk, these DALYs were multiplied by the PAF (the proportion by which the DALYs would be decreased in a specific year if the exposure to a risk factor in the past was equal to the TMREL) for the early-onset CRC risk-outcome pair for a given age-sex-location-year.

References

1. Global Burden of Disease Cancer Collaboration. Global, Regional, and National Cancer Incidence, Mortality, Years of Life Lost, Years Lived With Disability, and Disability-Adjusted Life-Years for 29 Cancer Groups, 1990 to 2017: A Systematic Analysis for the Global Burden of Disease Study. *JAMA Oncol* **2019**, *5*, 1749-1768, doi:10.1001/jamaoncol.2019.2996.
2. Foreman, K.J.; Lozano, R.; Lopez, A.D.; Murray, C.J. Modeling causes of death: an integrated approach using CODEm. *Popul Health Metr* **2012**, *10*, 1, doi:10.1186/1478-7954-10-1.
3. GBD 2019 Demographics Collaborators. Global age-sex-specific fertility, mortality, healthy life expectancy (HALE), and population estimates in 204 countries and territories, 1950-2019: a comprehensive demographic analysis for the Global Burden of Disease Study 2019. *Lancet* **2020**, *396*, 1160-1203, doi:10.1016/S0140-6736(20)30977-6.
4. GBD 2019 Risk Factors Collaborators. Global burden of 87 risk factors in 204

countries and territories, 1990-2019: a systematic analysis for the Global Burden of Disease Study 2019. *Lancet* **2020**, *396*, 1223-1249, doi:10.1016/S0140-6736(20)30752-2.
