# Supplementary material for: The Burden of Early-Onset Colorectal Cancer and Its Risk Factors from 1990 to 2019: A Systematic Analysis for the Global Burden of Disease Study 2019
Source: Cancers (Basel). 2022 Jul 19;14(14):3502. doi: 10.3390/cancers14143502 (PMC9323588; doi:10.3390/cancers14143502)
Supplement: Supplementary file 1 [file cancers-14-03502-s001.zip › Supplementary Tables.pdf]

Table S1. Incidence of early-onset colorectal cancer in 203 countries.

Country	2019						Change of incidence rate		
	Incidence number			Incidence rate (per 100 000)			2019 versus 1990		
	Value	LUI	UUI	Value	LUI	UUI	Value	LUI	UUI
Afghanistan	383.2	211.7	587.6	2.11	1.16	3.23	1.4%	-35.3%	78.4%
Albania	63.4	45.9	84.9	4.90	3.55	6.56	96.0%	38.3%	169.1%
Algeria	560.1	415.1	744.7	2.50	1.85	3.32	67.5%	13.8%	137.1%
American Samoa	1.5	1.1	2.0	5.32	3.86	7.22	42.8%	-3.7%	108.5%
Andorra	5.6	3.9	7.8	13.70	9.49	18.95	63.5%	-0.6%	156.1%
Angola	232.8	151.3	320.4	1.72	1.12	2.37	23.4%	-25.0%	100.8%
Antigua and Barbuda	2.0	1.6	2.5	4.25	3.44	5.32	63.2%	27.7%	108.2%
Argentina	1632.0	1226.0	2129.9	7.04	5.29	9.18	62.4%	20.8%	113.1%
Armenia	74.6	61.0	90.2	5.05	4.13	6.11	8.3%	-12.7%	31.9%
Australia	1306.2	981.0	1710.0	11.32	8.50	14.82	32.7%	-1.1%	73.8%
Austria	261.3	200.5	335.0	6.48	4.97	8.30	-36.5%	-51.8%	-17.6%
Azerbaijan	296.1	233.4	372.4	5.32	4.20	6.69	12.3%	-12.2%	42.5%
Bahamas	16.9	13.2	21.5	8.24	6.45	10.48	69.7%	28.0%	121.3%
Bahrain	38.5	28.5	50.2	4.16	3.08	5.43	117.7%	53.1%	201.2%
Bangladesh	919.7	590.0	1366.6	1.07	0.68	1.59	34.0%	-21.0%	131.1%
Barbados	12.1	9.5	15.2	8.61	6.81	10.87	78.7%	40.5%	129.5%
Belarus	341.2	251.5	462.1	7.83	5.77	10.60	13.8%	-17.0%	52.6%
Belgium	352.7	262.5	467.3	7.04	5.24	9.33	7.3%	-21.1%	42.3%
Belize	7.5	6.2	8.8	3.33	2.78	3.93	213.6%	151.1%	301.2%
Benin	65.6	44.4	93.5	1.13	0.76	1.61	21.1%	-19.1%	81.1%
Bermuda	2.9	2.2	3.7	10.28	7.93	13.38	54.0%	14.8%	105.4%
Bhutan	6.0	3.0	9.4	1.39	0.69	2.15	73.9%	-0.7%	219.5%
Bolivia	168.6	110.1	237.7	2.76	1.80	3.89	66.5%	3.0%	157.5%
Bosnia and Herzegovina	136.3	103.2	175.8	8.98	6.80	11.58	119.9%	62.4%	192.0%
Botswana	51.4	29.9	78.6	3.89	2.26	5.94	130.0%	25.4%	291.6%
Brazil	5615.3	5286.4	5915.3	4.86	4.58	5.12	97.2%	84.3%	110.6%
Brunei Darussalam	26.2	21.3	32.0	9.86	8.01	12.02	62.3%	17.2%	114.6%
Bulgaria	363.8	271.9	477.6	12.00	8.97	15.76	41.0%	4.9%	88.0%
Burkina Faso	116.8	81.3	160.3	1.14	0.79	1.57	34.3%	-8.3%	89.3%
Burundi	73.4	49.5	107.1	1.34	0.90	1.95	-12.1%	-45.8%	55.6%
Cabo Verde	6.4	4.7	8.8	2.10	1.53	2.86	121.1%	58.6%	214.4%
Cambodia	340.2	255.2	463.0	3.87	2.90	5.27	86.1%	20.2%	198.9%
Cameroon	250.3	162.5	363.7	1.74	1.13	2.53	23.3%	-23.3%	91.3%
Canada	1761.2	1308.8	2319.1	10.89	8.09	14.34	37.9%	1.4%	85.5%
Central African Republic	33.7	22.5	50.3	1.32	0.88	1.97	-6.3%	-39.3%	44.8%
Chad	68.8	48.7	94.6	1.01	0.71	1.38	29.0%	-11.6%	88.3%
Chile	477.8	355.4	624.0	5.20	3.86	6.79	115.2%	55.2%	187.3%
China	87382.8	72880.2	103288.4	12.12	10.11	14.33	238.1%	165.9%	325.9%
Colombia	1239.8	917.1	1642.9	4.98	3.68	6.60	122.4%	62.8%	196.5%
Comoros	7.0	4.0	10.1	1.87	1.07	2.71	49.6%	-22.7%	450.1%
Congo	64.5	40.2	95.6	2.41	1.50	3.57	13.5%	-35.1%	102.4%
Cook Islands	0.3	0.2	0.4	3.73	2.23	5.19	48.8%	-16.5%	143.5%
Costa Rica	191.1	144.6	251.2	7.66	5.79	10.06	183.8%	109.4%	277.3%
Croatia	189.1	138.0	248.8	10.14	7.41	13.35	37.3%	-0.6%	85.3%
Cuba	392.6	302.2	499.0	7.40	5.69	9.40	74.6%	33.9%	124.5%
Cyprus	41.5	33.2	50.4	6.02	4.81	7.30	126.9%	71.3%	194.2%
Czechia	428.7	324.8	550.4	8.93	6.76	11.46	-8.7%	-31.4%	18.1%
Cote d'Ivoire	198.6	133.9	276.0	1.52	1.03	2.11	13.9%	-28.6%	69.7%
Democratic People's Republic of Korea	862.4	537.6	1328.6	6.20	3.86	9.55	16.9%	-33.5%	100.5%
Democratic Republic of the Congo	424.2	271.4	635.3	1.02	0.65	1.52	-0.9%	-41.5%	52.0%
Denmark	202.2	149.0	266.1	7.89	5.81	10.38	12.7%	-17.3%	52.2%

Country	2019						Change of incidence rate		
	Incidence number			Incidence rate (per 100 000)			2019 versus 1990		
	Value	LUI	UUI	Value	LUI	UUI	Value	LUI	UUI
Djibouti	15.9	9.3	25.0	2.48	1.45	3.90	84.1%	3.5%	215.6%
Dominica	1.4	1.1	1.9	4.23	3.19	5.52	58.4%	13.2%	118.0%
Dominican Republic	262.7	179.0	360.4	4.55	3.10	6.25	170.3%	82.9%	282.1%
Ecuador	418.2	315.3	551.7	4.55	3.43	6.00	188.7%	111.1%	284.3%
Egypt	1359.9	946.0	1871.1	2.64	1.84	3.64	60.9%	10.5%	128.1%
El Salvador	139.5	100.6	190.6	4.25	3.07	5.81	201.8%	110.4%	309.5%
Equatorial Guinea	15.6	8.6	26.4	2.09	1.15	3.53	70.6%	-15.6%	203.7%
Eritrea	71.6	48.5	103.5	2.07	1.40	3.00	58.4%	-1.2%	161.6%
Estonia	51.8	38.0	68.3	8.90	6.54	11.74	37.1%	-2.5%	83.9%
Eswatini	16.7	9.5	25.8	2.77	1.56	4.27	91.3%	10.6%	212.6%
Ethiopia	711.0	518.1	987.0	1.35	0.99	1.88	-19.2%	-46.4%	53.9%
Fiji	15.4	11.5	20.3	3.30	2.46	4.36	40.1%	-5.9%	109.7%
Finland	141.1	105.0	188.6	6.05	4.50	8.08	3.6%	-24.4%	38.7%
France	2261.1	1644.4	3053.4	7.95	5.78	10.74	34.9%	-2.7%	81.3%
Gabon	26.9	17.2	39.0	2.91	1.86	4.22	17.1%	-34.0%	102.4%
Gambia	10.4	6.8	15.1	0.94	0.61	1.35	45.2%	-16.2%	137.2%
Georgia	112.5	90.2	138.5	6.80	5.45	8.37	17.2%	-10.0%	49.0%
Germany	3036.1	2283.3	4096.6	8.46	6.36	11.41	14.5%	-14.3%	55.8%
Ghana	317.6	219.9	437.1	1.91	1.32	2.63	56.0%	4.1%	138.4%
Greece	338.9	256.3	446.4	7.58	5.73	9.98	81.4%	34.2%	142.1%
Greenland	2.5	1.9	3.3	9.37	7.10	12.49	25.4%	-10.0%	82.5%
Grenada	2.9	2.3	3.5	5.33	4.33	6.47	78.2%	39.5%	125.0%
Guam	5.9	4.7	7.4	7.31	5.84	9.18	70.2%	25.1%	132.6%
Guatemala	279.4	213.0	360.3	2.92	2.23	3.76	150.9%	82.0%	229.5%
Guinea	62.3	44.5	84.8	1.09	0.78	1.48	23.4%	-18.2%	83.8%
Guinea-Bissau	15.2	11.0	20.5	1.60	1.16	2.17	7.0%	-29.3%	61.2%
Guyana	20.2	14.8	27.3	4.87	3.57	6.59	99.2%	41.0%	180.4%
Haiti	145.3	94.9	211.6	2.22	1.45	3.23	5.3%	-33.2%	62.5%
Honduras	85.4	51.3	131.2	1.65	0.99	2.54	25.0%	-25.4%	100.9%
Hungary	526.5	409.9	674.5	11.86	9.23	15.19	27.2%	-1.6%	63.5%
Iceland	9.2	7.7	10.9	5.65	4.69	6.70	22.1%	-3.0%	51.9%
India	14736.4	12218.4	17329.4	1.94	1.61	2.28	60.3%	25.8%	95.6%
Indonesia	6764.9	4855.1	8818.0	4.74	3.40	6.18	102.1%	53.1%	170.4%
Iran	1867.1	1719.9	2045.0	3.95	3.64	4.33	136.9%	85.6%	198.9%
Iraq	651.5	468.0	925.4	2.82	2.02	4.00	71.9%	0.8%	189.8%
Ireland	191.9	139.3	253.8	8.23	5.97	10.88	24.4%	-10.2%	67.0%
Israel	281.9	206.0	372.4	6.44	4.71	8.51	40.6%	2.5%	87.1%
Italy	2646.3	2110.0	3239.7	10.44	8.32	12.78	32.9%	6.6%	64.6%
Jamaica	87.1	63.4	115.6	5.69	4.14	7.55	263.4%	160.1%	396.2%
Japan	6649.6	5541.5	7919.8	12.85	10.71	15.30	22.4%	1.7%	45.1%
Jordan	250.4	201.7	310.4	3.95	3.18	4.90	74.8%	26.0%	134.7%
Kazakhstan	407.8	335.3	487.7	4.39	3.61	5.25	-1.5%	-18.3%	18.2%
Kenya	375.8	288.8	483.1	1.44	1.11	1.85	82.1%	35.2%	147.7%
Kiribati	2.1	1.5	2.9	3.48	2.55	4.72	4.6%	-29.2%	57.4%
Kuwait	96.9	77.0	121.0	3.33	2.65	4.16	95.9%	53.5%	152.9%
Kyrgyzstan	77.5	65.1	90.4	2.31	1.94	2.69	-36.2%	-47.9%	-23.1%
Lao People's Democratic Republic	141.6	91.3	195.6	3.63	2.34	5.02	37.6%	-13.5%	134.3%
Latvia	53.3	41.3	68.8	6.50	5.05	8.40	7.1%	-18.5%	39.6%
Lebanon	182.9	133.0	239.7	6.85	4.98	8.97	121.1%	36.7%	238.3%
Lesotho	26.4	16.5	37.8	2.30	1.44	3.30	156.7%	38.9%	333.1%
Liberia	27.7	16.5	41.6	1.12	0.67	1.68	4.1%	-38.6%	58.8%
Libya	210.1	147.5	292.1	5.11	3.59	7.11	84.5%	-1.8%	220.5%

Country	2019						Change of incidence rate		
	Incidence number			Incidence rate (per 100 000)			2019 versus 1990		
	Value	LUI	UUI	Value	LUI	UUI	Value	LUI	UUI
Lithuania	76.0	57.7	97.8	6.27	4.76	8.07	6.5%	-20.0%	35.5%
Luxembourg	21.4	17.0	26.5	6.96	5.54	8.62	-8.9%	-28.9%	14.3%
Madagascar	195.8	133.4	272.9	1.48	1.01	2.07	-0.2%	-35.4%	48.5%
Malawi	84.8	56.1	122.8	0.94	0.62	1.36	23.3%	-21.5%	92.3%
Malaysia	979.6	699.5	1319.1	5.61	4.01	7.56	83.8%	24.6%	163.2%
Maldives	7.9	6.3	10.1	2.49	1.97	3.16	54.6%	-2.3%	184.6%
Mali	115.9	79.7	158.3	1.20	0.82	1.64	5.9%	-27.0%	52.1%
Malta	12.8	10.2	15.7	6.50	5.17	7.98	62.1%	24.6%	109.3%
Marshall Islands	1.3	0.8	1.7	4.13	2.77	5.76	68.2%	12.3%	138.1%
Mauritania	21.6	13.8	31.5	1.12	0.71	1.63	-8.4%	-45.5%	55.8%
Mauritius	35.9	27.6	45.6	5.52	4.24	7.02	151.2%	89.5%	228.4%
Mexico	2943.1	2499.8	3432.6	4.44	3.77	5.18	179.4%	136.3%	226.2%
Micronesia	2.3	0.8	3.4	4.27	1.45	6.43	39.7%	-53.3%	120.3%
Monaco	2.1	1.5	2.9	14.72	10.63	20.06	37.9%	-10.1%	105.9%
Mongolia	60.4	44.5	82.2	3.34	2.46	4.55	83.8%	28.4%	163.8%
Montenegro	19.2	15.2	23.5	6.48	5.12	7.95	35.8%	1.7%	75.0%
Morocco	409.3	282.0	612.9	2.14	1.47	3.20	81.7%	16.3%	189.8%
Mozambique	173.8	110.4	251.9	1.30	0.83	1.89	72.9%	9.6%	161.1%
Myanmar	1219.3	823.4	1700.3	4.22	2.85	5.88	69.7%	2.6%	158.0%
Namibia	20.4	12.6	31.2	1.63	1.00	2.48	81.2%	4.1%	220.6%
Nauru	0.4	0.2	0.5	6.41	3.65	8.96	4.9%	-28.9%	52.2%
Nepal	172.6	113.5	250.9	1.07	0.70	1.55	28.4%	-24.6%	108.2%
Netherlands	769.1	570.9	1002.1	10.25	7.61	13.35	36.1%	1.1%	78.6%
New Zealand	214.2	170.8	261.8	10.79	8.60	13.19	11.4%	-12.3%	40.1%
Nicaragua	98.4	74.6	125.0	2.81	2.13	3.57	99.5%	40.2%	175.6%
Niger	69.0	45.0	103.4	0.73	0.48	1.09	-11.6%	-41.5%	27.2%
Nigeria	1034.4	719.0	1452.7	1.03	0.71	1.44	28.3%	-14.4%	92.6%
Niue	0.0	0.0	0.1	6.00	3.51	9.30	40.2%	-23.2%	133.5%
North Macedonia	88.4	67.0	115.9	8.15	6.18	10.69	88.8%	40.2%	151.6%
Northern Mariana Islands	1.8	1.3	2.5	8.96	6.36	12.30	32.1%	-19.9%	118.4%
Norway	217.3	177.6	261.0	8.72	7.13	10.48	18.8%	-3.2%	44.2%
Oman	65.5	47.7	98.4	2.09	1.53	3.14	55.8%	-11.1%	159.6%
Pakistan	2418.7	1820.5	3066.7	2.16	1.63	2.74	87.5%	35.0%	161.8%
Palau	0.6	0.4	0.8	6.04	4.18	8.26	72.3%	3.9%	172.2%
Palestine	108.5	88.7	130.7	4.28	3.50	5.15	45.9%	-4.3%	132.9%
Panama	94.2	66.8	125.9	4.48	3.18	5.99	114.3%	50.0%	193.8%
Papua New Guinea	102.9	71.4	144.2	2.02	1.40	2.84	32.7%	-5.7%	92.0%
Paraguay	139.6	100.0	189.5	3.75	2.69	5.10	165.3%	80.7%	275.0%
Peru	920.5	649.2	1271.4	5.16	3.64	7.13	146.8%	65.0%	260.7%
Philippines	3354.3	2684.6	4197.0	5.74	4.59	7.18	36.5%	7.9%	77.9%
Poland	1319.1	1088.7	1590.6	7.25	5.98	8.74	43.0%	17.5%	72.5%
Portugal	647.6	466.4	857.5	13.80	9.94	18.28	91.7%	38.3%	159.7%
Puerto Rico	158.1	113.8	216.4	9.98	7.18	13.65	103.5%	44.3%	183.5%
Qatar	59.3	42.8	80.7	2.75	1.98	3.74	74.2%	7.3%	175.0%
Republic of Korea	2408.4	1928.5	2950.4	9.25	7.40	11.33	139.1%	90.5%	197.8%
Republic of Moldova	129.6	106.6	155.1	7.05	5.80	8.44	5.2%	-13.9%	27.0%
Romania	886.0	701.8	1108.8	10.23	8.11	12.81	109.5%	64.6%	163.4%
Russian Federation	6551.4	5526.0	7685.3	9.57	8.07	11.22	81.8%	56.0%	111.4%
Rwanda	109.0	73.2	158.4	1.68	1.13	2.45	0.8%	-41.1%	92.0%
Saint Kitts and Nevis	1.6	0.5	2.5	5.09	1.73	7.92	20.1%	-58.5%	93.6%
Saint Lucia	4.5	3.6	5.5	4.80	3.89	5.89	96.9%	54.3%	145.6%
Saint Vincent and the Grenadines	2.9	2.4	3.5	5.15	4.26	6.18	112.0%	69.3%	161.7%

Country	2019						Change of incidence rate		
	Incidence number			Incidence rate (per 100 000)			2019 versus 1990		
	Value	LUI	UUI	Value	LUI	UUI	Value	LUI	UUI
Samoa	2.9	1.8	4.1	2.80	1.75	3.98	44.7%	-15.4%	137.5%
San Marino	1.5	1.0	2.1	9.66	6.81	13.57	59.3%	7.5%	136.1%
Sao Tome and Principe	2.7	1.7	4.0	2.50	1.57	3.70	108.7%	23.3%	265.7%
Saudi Arabia	1146.9	810.1	1598.2	4.71	3.33	6.56	305.3%	134.8%	571.5%
Senegal	94.5	61.0	132.2	1.29	0.83	1.80	16.4%	-26.3%	74.3%
Serbia	435.1	328.4	566.0	10.87	8.20	14.14	53.4%	8.0%	117.7%
Seychelles	5.7	4.7	6.8	10.45	8.58	12.47	163.6%	108.9%	235.5%
Sierra Leone	44.1	30.0	61.7	1.06	0.72	1.49	19.7%	-22.2%	81.3%
Singapore	210.2	160.3	272.4	6.83	5.21	8.86	2.6%	-23.0%	35.0%
Slovakia	310.4	225.6	419.6	11.80	8.57	15.95	48.5%	6.9%	101.4%
Slovenia	84.5	61.6	116.0	9.43	6.87	12.95	32.3%	-16.3%	100.9%
Solomon Islands	14.5	8.1	20.5	4.43	2.46	6.23	57.0%	5.1%	124.9%
Somalia	88.5	48.5	169.2	0.93	0.51	1.79	-13.6%	-53.5%	44.9%
South Africa	789.4	675.0	913.0	2.59	2.22	3.00	4.6%	-15.3%	28.2%
South Sudan	70.3	41.7	115.6	1.67	0.99	2.74	16.0%	-26.8%	96.1%
Spain	2274.7	1665.4	3048.3	11.01	8.06	14.75	54.2%	12.4%	106.0%
Sri Lanka	283.5	198.8	392.6	2.60	1.82	3.60	91.7%	28.5%	182.2%
Sudan	365.9	214.0	574.0	1.76	1.03	2.75	58.9%	-12.4%	159.4%
Suriname	15.4	11.9	19.4	5.33	4.15	6.73	97.6%	49.1%	175.4%
Sweden	320.3	264.6	382.6	7.15	5.91	8.54	13.2%	-7.7%	37.3%
Switzerland	262.4	195.6	345.2	6.57	4.90	8.64	16.7%	-15.0%	56.7%
Syrian Arab Republic	174.0	124.6	240.7	2.34	1.68	3.24	40.6%	-12.9%	123.3%
Tajikistan	146.3	113.3	186.6	2.94	2.28	3.76	-24.8%	-43.0%	-2.1%
Thailand	2304.6	1613.1	3170.4	6.46	4.52	8.88	109.3%	45.1%	199.8%
Timor-Leste	17.1	5.5	24.7	2.62	0.84	3.79	65.1%	-57.4%	175.4%
Togo	54.9	35.2	79.1	1.40	0.90	2.01	32.7%	-12.8%	92.7%
Tokelau	0.0	0.0	0.0	4.06	2.33	6.03	58.7%	-8.9%	155.2%
Tonga	0.9	0.6	1.2	1.76	1.23	2.47	49.7%	-1.9%	127.1%
Trinidad and Tobago	40.2	27.7	55.3	5.79	3.99	7.96	58.0%	9.7%	120.0%
Tunisia	212.5	147.7	293.0	3.52	2.45	4.85	146.6%	56.3%	265.3%
Turkey	2428.7	1888.4	3120.2	5.32	4.14	6.83	50.6%	2.6%	116.1%
Turkmenistan	77.2	60.7	100.0	2.93	2.30	3.80	0.8%	-21.6%	32.6%
Tuvalu	0.2	0.1	0.3	3.72	2.43	5.23	23.9%	-19.5%	86.7%
Uganda	389.8	277.5	541.7	2.06	1.47	2.87	120.9%	42.6%	250.8%
Ukraine	2035.5	1663.2	2476.0	9.79	8.00	11.91	12.4%	-9.5%	39.1%
United Arab Emirates	289.3	191.3	409.1	4.15	2.75	5.87	102.5%	8.1%	267.4%
United Kingdom	2707.0	2215.2	3273.0	8.88	7.27	10.74	28.8%	4.8%	56.0%
United Republic of Tanzania	482.1	327.0	702.2	1.81	1.23	2.63	42.3%	-3.7%	106.6%
United States of America	16735.3	14188.0	19774.1	11.12	9.42	13.13	41.7%	20.0%	68.5%
United States Virgin Islands	4.2	3.0	6.0	9.78	6.94	13.74	60.8%	9.1%	140.1%
Uruguay	123.4	92.0	163.8	7.49	5.58	9.94	24.8%	-6.9%	68.7%
Uzbekistan	578.4	480.5	693.0	3.20	2.66	3.83	11.4%	-8.7%	34.9%
Vanuatu	3.9	2.5	5.6	2.69	1.72	3.83	46.2%	-11.8%	134.6%
Venezuela	711.3	514.3	957.4	4.94	3.57	6.65	133.0%	68.8%	218.3%
Viet Nam	3496.8	2485.0	4829.1	6.59	4.69	9.11	313.3%	159.4%	545.5%
Yemen	229.3	150.1	326.9	1.47	0.96	2.09	41.4%	-20.9%	163.2%
Zambia	237.8	158.8	338.5	2.63	1.76	3.75	49.6%	-4.9%	124.4%
Zimbabwe	206.8	139.8	288.0	2.75	1.86	3.83	94.3%	24.4%	183.5%

95% UI, 95% uncertainty intervals; LUI, lower uncertainty interval; UUI, upper uncertainty interval.

Table S2. Deaths of early-onset colorectal cancer in 203 countries.

Country	2019						Change of death rate		
	Death number			Death rate (per 100 000)			2019 versus 1990		
	Value	LUI	UUI	Value	LUI	UUI	Value	LUI	UUI
Afghanistan	302.0	165.3	462.7	1.66	0.91	2.54	-3.9%	-38.9%	70.4%
Albania	24.1	17.5	32.6	1.86	1.35	2.52	27.4%	-8.4%	73.7%
Algeria	286.8	211.3	381.3	1.28	0.94	1.70	27.5%	-14.1%	81.1%
American Samoa	0.8	0.6	1.1	3.01	2.19	4.08	29.6%	-12.7%	88.4%
Andorra	1.4	1.0	1.9	3.41	2.38	4.65	15.5%	-29.6%	82.7%
Angola	174.0	114.9	240.0	1.29	0.85	1.77	13.2%	-32.0%	81.4%
Antigua and Barbuda	0.9	0.7	1.1	1.83	1.48	2.30	29.0%	0.7%	65.1%
Argentina	781.7	706.7	865.8	3.37	3.05	3.73	23.9%	9.6%	39.7%
Armenia	34.2	27.8	41.1	2.32	1.88	2.78	-17.4%	-33.6%	1.3%
Australia	282.2	253.6	315.5	2.45	2.20	2.73	-14.4%	-23.6%	-3.3%
Austria	62.1	54.7	70.6	1.54	1.35	1.75	-57.3%	-62.8%	-50.9%
Azerbaijan	162.3	127.8	204.3	2.92	2.30	3.67	-5.3%	-26.0%	19.7%
Bahamas	8.2	6.4	10.4	4.00	3.12	5.07	44.6%	9.1%	88.6%
Bahrain	15.8	11.6	20.5	1.71	1.26	2.22	41.0%	-0.8%	96.2%
Bangladesh	604.9	388.1	899.5	0.70	0.45	1.04	10.5%	-34.1%	92.6%
Barbados	5.1	4.0	6.4	3.62	2.86	4.58	41.2%	10.4%	81.1%
Belarus	118.2	87.7	159.5	2.71	2.01	3.66	-23.1%	-43.5%	2.9%
Belgium	92.9	81.6	104.6	1.85	1.63	2.09	-27.4%	-37.0%	-16.6%
Belize	3.9	3.3	4.7	1.76	1.48	2.09	165.7%	115.6%	238.5%
Benin	48.7	32.8	68.7	0.84	0.56	1.18	14.9%	-24.3%	69.2%
Bermuda	0.8	0.6	1.0	2.86	2.22	3.71	-7.3%	-32.2%	22.8%
Bhutan	3.8	1.9	5.9	0.87	0.43	1.35	37.2%	-21.1%	150.3%
Bolivia	89.5	58.7	126.4	1.46	0.96	2.07	24.1%	-22.9%	91.6%
Bosnia and Herzegovina	57.8	44.1	74.6	3.81	2.90	4.92	57.3%	16.2%	109.4%
Botswana	31.9	18.6	48.4	2.41	1.41	3.66	96.0%	7.7%	237.2%
Brazil	2784.9	2629.9	2928.3	2.41	2.28	2.54	51.3%	41.4%	61.2%
Brunei Darussalam	11.1	9.1	13.5	4.19	3.43	5.06	24.3%	-8.6%	63.4%
Bulgaria	155.3	116.9	202.8	5.13	3.86	6.69	13.4%	-15.5%	51.1%
Burkina Faso	87.4	61.1	119.6	0.85	0.60	1.17	26.5%	-14.2%	81.8%
Burundi	56.4	37.9	82.8	1.03	0.69	1.51	-15.1%	-48.8%	50.5%
Cabo Verde	3.7	2.6	5.0	1.19	0.86	1.62	77.7%	27.5%	153.4%
Cambodia	213.8	161.3	289.7	2.43	1.84	3.30	52.7%	-1.5%	146.8%
Cameroon	180.0	117.3	261.9	1.25	0.82	1.82	13.1%	-30.7%	77.3%
Canada	362.6	323.0	406.4	2.24	2.00	2.51	-3.3%	-14.6%	9.7%
Central African Republic	27.0	17.9	40.5	1.05	0.70	1.58	-5.0%	-38.7%	47.4%
Chad	53.3	37.3	74.1	0.78	0.55	1.08	24.8%	-14.4%	82.3%
Chile	184.3	163.4	207.1	2.00	1.78	2.25	41.5%	22.5%	62.4%
China	26274.2	21892.4	31077.2	3.65	3.04	4.31	64.1%	29.5%	105.6%
Colombia	490.1	360.8	648.5	1.97	1.45	2.60	48.9%	10.4%	97.7%
Comoros	5.2	3.0	7.6	1.40	0.81	2.04	39.7%	-28.6%	407.2%
Congo	48.2	29.9	71.7	1.80	1.12	2.68	8.2%	-38.4%	94.6%
Cook Islands	0.1	0.1	0.2	1.53	0.91	2.12	16.8%	-34.7%	92.9%
Costa Rica	68.7	51.5	90.0	2.75	2.06	3.61	116.4%	58.6%	189.5%
Croatia	59.9	44.5	78.5	3.22	2.39	4.21	-2.2%	-29.3%	31.2%
Cuba	140.0	108.5	174.9	2.64	2.04	3.29	30.8%	0.8%	67.0%
Cyprus	9.9	8.2	12.0	1.44	1.18	1.73	18.7%	-10.3%	51.4%
Czechia	137.5	105.3	175.1	2.86	2.19	3.65	-40.4%	-55.3%	-22.8%
Cote d'Ivoire	147.8	99.4	205.5	1.13	0.76	1.57	9.7%	-31.4%	62.8%
Democratic People's Republic of Korea	472.7	296.9	723.9	3.40	2.13	5.20	10.7%	-37.8%	90.5%
Democratic Republic of the Congo	325.9	205.7	485.7	0.78	0.49	1.16	-4.1%	-44.6%	50.3%
Denmark	53.8	47.2	60.6	2.10	1.84	2.36	-31.8%	-41.4%	-21.9%

Country	2019						Change of death rate		
	Death number			Death rate (per 100 000)			2019 versus 1990		
	Value	LUI	UUI	Value	LUI	UUI	Value	LUI	UUI
Djibouti	11.6	6.8	18.3	1.81	1.05	2.86	71.8%	-2.9%	191.3%
Dominica	0.8	0.6	1.0	2.28	1.73	2.99	48.2%	4.0%	105.2%
Dominican Republic	139.2	94.8	193.6	2.41	1.64	3.36	113.6%	43.3%	202.3%
Ecuador	169.7	127.8	223.2	1.85	1.39	2.43	87.5%	36.6%	147.1%
Egypt	765.9	532.6	1058.6	1.49	1.04	2.06	24.6%	-14.1%	75.3%
El Salvador	65.2	47.0	88.5	1.99	1.43	2.70	112.9%	50.1%	191.6%
Equatorial Guinea	10.5	5.8	17.7	1.40	0.77	2.36	41.2%	-30.3%	158.9%
Eritrea	55.7	37.7	80.7	1.61	1.09	2.34	51.1%	-6.7%	149.0%
Estonia	14.5	10.6	19.3	2.49	1.82	3.31	-18.0%	-41.4%	11.1%
Eswatini	12.0	6.9	18.6	1.98	1.14	3.07	80.2%	4.3%	196.5%
Ethiopia	525.4	382.3	728.0	1.00	0.73	1.39	-26.9%	-52.5%	40.7%
Fiji	9.7	7.2	12.8	2.08	1.54	2.74	29.7%	-12.6%	95.4%
Finland	34.4	29.9	39.2	1.47	1.28	1.68	-29.5%	-40.6%	-18.1%
France	584.1	510.8	658.0	2.05	1.80	2.31	-13.8%	-25.7%	-1.8%
Gabon	18.6	11.8	26.9	2.02	1.28	2.92	7.0%	-40.6%	84.4%
Gambia	7.6	5.0	11.0	0.68	0.45	0.99	37.3%	-21.4%	126.7%
Georgia	58.4	46.7	72.0	3.53	2.82	4.35	10.1%	-15.2%	39.8%
Germany	790.9	708.5	883.8	2.20	1.97	2.46	-23.3%	-32.1%	-13.6%
Ghana	216.5	149.7	297.5	1.30	0.90	1.79	41.9%	-4.7%	118.9%
Greece	93.2	84.2	102.7	2.08	1.88	2.30	37.7%	22.3%	53.7%
Greenland	1.2	0.9	1.6	4.54	3.48	6.04	-1.0%	-29.8%	43.1%
Grenada	1.5	1.2	1.8	2.70	2.18	3.26	45.9%	13.3%	83.9%
Guam	2.9	2.3	3.6	3.52	2.80	4.43	60.1%	17.7%	117.1%
Guatemala	159.3	121.9	205.8	1.67	1.27	2.15	96.5%	46.2%	166.0%
Guinea	48.1	34.1	65.8	0.84	0.60	1.15	17.3%	-23.5%	75.9%
Guinea-Bissau	11.5	8.3	15.5	1.21	0.88	1.64	2.5%	-33.3%	55.8%
Guyana	12.3	9.0	16.5	2.96	2.16	3.97	76.6%	24.3%	149.4%
Haiti	105.5	68.6	154.2	1.61	1.05	2.35	-2.9%	-38.6%	48.1%
Honduras	49.7	29.7	76.2	0.96	0.57	1.47	3.7%	-38.2%	66.2%
Hungary	187.2	146.1	236.2	4.22	3.29	5.32	-10.9%	-30.1%	13.3%
Iceland	2.1	1.8	2.5	1.31	1.11	1.53	-11.6%	-28.3%	7.1%
India	9962.0	8305.9	11871.9	1.31	1.09	1.56	38.1%	9.2%	68.0%
Indonesia	4190.4	2996.4	5533.4	2.93	2.10	3.88	71.5%	28.5%	130.1%
Iran	829.9	769.4	899.6	1.76	1.63	1.90	68.4%	32.4%	110.7%
Iraq	350.6	252.0	492.8	1.52	1.09	2.13	33.2%	-21.5%	122.8%
Ireland	45.4	38.8	52.9	1.95	1.67	2.27	-26.9%	-38.6%	-13.5%
Israel	83.5	74.1	93.9	1.91	1.69	2.15	-8.9%	-20.6%	4.4%
Italy	595.0	558.1	628.7	2.35	2.20	2.48	-11.9%	-17.3%	-6.5%
Jamaica	41.2	30.5	54.8	2.69	1.99	3.58	204.3%	119.6%	311.5%
Japan	1426.9	1342.3	1480.2	2.76	2.59	2.86	-20.3%	-24.3%	-17.2%
Jordan	113.4	92.2	139.8	1.79	1.45	2.21	18.5%	-13.8%	58.3%
Kazakhstan	196.6	162.5	233.8	2.12	1.75	2.52	-22.4%	-35.5%	-6.8%
Kenya	278.3	211.6	356.9	1.07	0.81	1.37	82.9%	35.4%	145.0%
Kiribati	1.5	1.1	2.1	2.56	1.86	3.47	0.6%	-32.5%	51.7%
Kuwait	33.0	26.6	40.9	1.13	0.92	1.41	35.2%	7.6%	72.6%
Kyrgyzstan	42.3	35.5	49.9	1.26	1.06	1.48	-45.7%	-55.6%	-34.1%
Lao People's Democratic Republic	96.7	62.7	134.1	2.48	1.61	3.44	18.8%	-25.3%	102.1%
Latvia	20.4	15.9	26.3	2.49	1.94	3.20	-22.7%	-40.5%	-0.5%
Lebanon	67.7	49.8	88.9	2.53	1.86	3.33	29.0%	-20.2%	98.8%
Lesotho	19.5	12.2	28.2	1.70	1.07	2.46	146.9%	33.8%	320.5%
Liberia	20.6	12.3	31.3	0.83	0.50	1.27	-3.1%	-42.1%	47.6%
Libya	114.7	79.6	160.7	2.79	1.94	3.91	54.4%	-16.8%	167.9%

Country	2019						Change of death rate		
	Death number			Death rate (per 100 000)			2019 versus 1990		
	Value	LUI	UUI	Value	LUI	UUI	Value	LUI	UUI
Lithuania	30.4	23.3	38.8	2.51	1.92	3.20	-15.8%	-35.5%	6.7%
Luxembourg	5.4	4.7	6.3	1.77	1.52	2.05	-42.4%	-52.3%	-30.9%
Madagascar	151.4	103.3	211.9	1.15	0.78	1.60	-2.6%	-37.3%	45.2%
Malawi	63.3	41.7	91.7	0.70	0.46	1.02	17.3%	-25.8%	82.0%
Malaysia	442.0	314.7	591.7	2.53	1.80	3.39	28.7%	-11.7%	84.1%
Maldives	3.1	2.5	3.9	0.99	0.78	1.24	-11.7%	-43.9%	64.5%
Mali	86.9	60.1	118.7	0.90	0.62	1.23	-2.1%	-33.3%	43.3%
Malta	3.4	2.9	4.0	1.73	1.45	2.04	4.9%	-14.8%	28.4%
Marshall Islands	0.9	0.6	1.2	2.86	1.91	3.97	60.3%	7.2%	126.1%
Mauritania	15.0	9.7	21.8	0.77	0.50	1.13	-19.4%	-52.1%	37.6%
Mauritius	16.1	12.5	20.5	2.48	1.92	3.16	98.6%	49.6%	158.9%
Mexico	1334.6	1131.1	1553.6	2.01	1.71	2.34	109.0%	77.2%	144.9%
Micronesia	1.5	0.5	2.2	2.74	0.90	4.13	21.9%	-60.3%	93.0%
Monaco	0.5	0.4	0.7	3.60	2.65	5.01	3.5%	-30.7%	55.3%
Mongolia	37.6	27.6	50.9	2.08	1.52	2.82	55.4%	8.4%	122.9%
Montenegro	6.9	5.5	8.5	2.34	1.86	2.88	9.2%	-17.8%	42.2%
Morocco	252.3	174.3	383.0	1.32	0.91	2.00	50.6%	-4.1%	138.0%
Mozambique	132.2	83.3	192.3	0.99	0.63	1.44	59.8%	0.7%	147.1%
Myanmar	787.0	531.8	1105.6	2.72	1.84	3.82	42.5%	-11.6%	121.5%
Namibia	13.7	8.5	20.8	1.09	0.68	1.66	56.3%	-10.9%	178.4%
Nauru	0.2	0.1	0.3	3.64	2.07	5.01	-7.3%	-37.5%	35.8%
Nepal	118.1	77.0	172.2	0.73	0.48	1.06	9.4%	-35.4%	77.8%
Netherlands	181.4	160.4	204.0	2.42	2.14	2.72	-7.5%	-19.3%	5.5%
New Zealand	54.9	49.5	61.2	2.77	2.49	3.08	-21.9%	-31.9%	-10.2%
Nicaragua	44.2	33.4	56.2	1.26	0.95	1.60	41.9%	0.0%	95.2%
Niger	52.6	34.7	78.4	0.55	0.37	0.83	-16.9%	-44.7%	20.6%
Nigeria	748.3	514.4	1052.5	0.74	0.51	1.04	18.4%	-21.5%	84.8%
Niue	0.0	0.0	0.0	2.77	1.62	4.32	8.9%	-40.2%	82.1%
North Macedonia	36.9	28.1	48.3	3.40	2.60	4.45	34.0%	0.4%	78.9%
Northern Mariana Islands	0.8	0.6	1.1	3.83	2.70	5.20	12.2%	-32.4%	84.8%
Norway	53.8	48.9	57.5	2.16	1.96	2.31	-22.7%	-29.7%	-17.0%
Oman	25.0	18.5	36.9	0.80	0.59	1.18	-3.9%	-45.1%	56.9%
Pakistan	1732.1	1336.3	2215.2	1.55	1.20	1.98	69.9%	22.5%	139.2%
Palau	0.3	0.2	0.4	2.79	1.91	3.81	43.8%	-12.6%	130.8%
Palestine	57.0	46.4	68.4	2.25	1.83	2.69	17.2%	-22.9%	85.6%
Panama	38.9	27.7	51.6	1.85	1.32	2.45	59.6%	12.0%	117.0%
Papua New Guinea	73.0	50.4	102.7	1.44	0.99	2.02	27.3%	-10.1%	85.6%
Paraguay	72.0	51.5	97.4	1.94	1.39	2.62	107.5%	41.9%	196.5%
Peru	266.5	188.2	371.1	1.50	1.06	2.08	33.9%	-10.0%	95.1%
Philippines	2015.4	1636.9	2510.6	3.45	2.80	4.29	23.1%	-3.6%	57.3%
Poland	567.8	462.7	678.9	3.12	2.54	3.73	-2.7%	-21.0%	15.7%
Portugal	156.6	137.9	177.4	3.34	2.94	3.78	9.7%	-4.9%	26.6%
Puerto Rico	49.2	35.4	67.0	3.10	2.23	4.23	37.8%	-2.1%	91.6%
Qatar	20.2	14.6	27.4	0.93	0.68	1.27	-4.4%	-40.5%	47.5%
Republic of Korea	567.8	497.6	646.2	2.18	1.91	2.48	6.3%	-8.5%	24.6%
Republic of Moldova	59.5	49.3	70.4	3.24	2.69	3.83	-17.4%	-31.7%	-1.5%
Romania	359.2	285.9	452.1	4.15	3.30	5.22	45.5%	14.6%	83.1%
Russian Federation	2240.2	1883.2	2619.9	3.27	2.75	3.83	30.2%	11.1%	51.5%
Rwanda	79.1	52.8	114.6	1.22	0.82	1.77	-8.2%	-46.7%	76.4%
Saint Kitts and Nevis	0.7	0.2	1.1	2.17	0.73	3.40	-5.7%	-67.9%	49.9%
Saint Lucia	2.2	1.7	2.7	2.32	1.87	2.84	56.5%	22.9%	94.9%
Saint Vincent and the Grenadines	1.6	1.3	1.9	2.76	2.27	3.32	90.6%	52.7%	132.9%

Country	2019						Change of death rate 2019 versus 1990		
	Death number			Death rate (per 100 000)					
	Value	LUI	UUI	Value	LUI	UUI	Value	LUI	UUI
Samoa	1.7	1.1	2.4	1.64	1.02	2.35	28.3%	-23.8%	112.3%
San Marino	0.4	0.2	0.6	2.43	1.44	4.00	20.2%	-33.0%	116.2%
Sao Tome and Principe	1.8	1.1	2.6	1.67	1.05	2.47	81.9%	8.2%	213.6%
Saudi Arabia	495.1	357.0	685.8	2.03	1.47	2.82	116.1%	23.1%	278.4%
Senegal	69.3	45.0	96.4	0.94	0.61	1.31	9.2%	-30.4%	62.5%
Serbia	152.9	115.6	198.8	3.82	2.89	4.97	-0.1%	-29.0%	39.2%
Seychelles	2.8	2.3	3.4	5.14	4.22	6.21	103.9%	61.1%	158.6%
Sierra Leone	32.8	22.2	46.1	0.79	0.53	1.11	12.2%	-27.5%	70.3%
Singapore	50.4	44.3	57.1	1.64	1.44	1.86	-42.3%	-50.4%	-33.1%
Slovakia	94.4	68.5	126.5	3.59	2.60	4.81	-2.4%	-29.4%	33.3%
Slovenia	21.5	15.6	28.8	2.40	1.74	3.21	-18.6%	-47.8%	23.3%
Solomon Islands	9.7	5.3	13.7	2.95	1.62	4.16	43.2%	-4.2%	105.3%
Somalia	74.7	44.1	142.0	0.79	0.47	1.50	-12.4%	-48.2%	46.2%
South Africa	509.9	439.6	590.6	1.67	1.44	1.94	-5.7%	-23.1%	16.8%
South Sudan	56.5	32.7	94.4	1.34	0.78	2.24	15.5%	-29.2%	98.6%
Spain	535.5	477.7	600.6	2.59	2.31	2.91	1.2%	-11.6%	14.7%
Sri Lanka	117.1	83.2	162.6	1.07	0.76	1.49	27.0%	-14.7%	83.5%
Sudan	229.6	134.0	359.9	1.10	0.64	1.73	28.1%	-27.8%	113.2%
Suriname	8.7	6.8	11.0	3.03	2.36	3.82	70.7%	28.5%	137.2%
Sweden	89.5	81.1	98.2	2.00	1.81	2.19	-16.2%	-25.4%	-7.0%
Switzerland	60.6	53.0	68.6	1.52	1.33	1.72	-19.3%	-30.9%	-7.0%
Syrian Arab Republic	87.9	63.3	122.8	1.18	0.85	1.65	6.6%	-33.5%	70.2%
Tajikistan	92.4	71.2	118.4	1.86	1.43	2.38	-29.1%	-46.4%	-8.4%
Thailand	964.8	679.9	1314.7	2.70	1.91	3.68	38.0%	-4.3%	100.6%
Timor-Leste	11.2	3.5	16.3	1.72	0.54	2.50	39.1%	-65.4%	135.7%
Togo	40.2	25.6	57.5	1.02	0.65	1.46	26.6%	-18.1%	84.0%
Tokelau	0.0	0.0	0.0	2.23	1.28	3.33	27.2%	-27.8%	104.4%
Tonga	0.5	0.4	0.7	1.05	0.73	1.49	34.0%	-12.5%	103.0%
Trinidad and Tobago	19.4	13.5	26.6	2.79	1.94	3.82	29.3%	-9.7%	78.3%
Tunisia	96.0	66.6	133.3	1.59	1.10	2.21	73.7%	10.3%	159.7%
Turkey	1022.2	799.7	1297.6	2.24	1.75	2.84	-9.0%	-37.0%	30.9%
Turkmenistan	42.7	33.4	55.6	1.62	1.27	2.11	-14.1%	-33.4%	12.6%
Tuvalu	0.1	0.1	0.2	2.31	1.51	3.27	6.9%	-30.5%	59.6%
Uganda	282.4	199.2	393.4	1.50	1.05	2.08	104.4%	31.4%	228.9%
Ukraine	975.6	796.4	1203.8	4.69	3.83	5.79	-7.5%	-25.7%	15.3%
United Arab Emirates	153.9	102.6	217.5	2.21	1.47	3.12	61.5%	-13.1%	189.9%
United Kingdom	707.6	686.7	728.6	2.32	2.25	2.39	-15.4%	-18.5%	-12.5%
United Republic of Tanzania	352.0	237.3	517.4	1.32	0.89	1.94	34.8%	-9.2%	97.3%
United States of America	4180.7	4040.5	4334.6	2.78	2.68	2.88	14.0%	9.0%	20.5%
United States Virgin Islands	1.9	1.4	2.7	4.38	3.16	6.20	29.2%	-12.1%	90.6%
Uruguay	55.4	49.2	62.1	3.36	2.99	3.77	-2.6%	-16.8%	13.1%
Uzbekistan	316.0	262.6	379.4	1.75	1.45	2.10	-2.4%	-19.6%	18.7%
Vanuatu	2.8	1.8	4.0	1.91	1.22	2.73	39.8%	-16.1%	124.6%
Venezuela	313.3	227.1	421.9	2.18	1.58	2.93	71.9%	24.3%	134.0%
Viet Nam	1676.0	1192.4	2318.0	3.16	2.25	4.37	188.7%	81.1%	350.5%
Yemen	160.6	104.7	231.7	1.03	0.67	1.48	26.2%	-29.1%	130.2%
Zambia	171.9	114.3	244.2	1.90	1.27	2.71	37.0%	-14.2%	108.2%
Zimbabwe	151.0	101.5	211.3	2.01	1.35	2.81	100.2%	27.6%	193.0%

95% UI, 95% uncertainty intervals; LUI, lower uncertainty interval; UUI, upper uncertainty interval.

Table S3. Disability-adjusted life-years of early-onset colorectal cancer in 203 countries.

Country	2019						Change of death rate		
	DALYs			DALY rate (per 100 000)			2019 versus 1990		
	Value	LUI	UUI	Value	LUI	UUI	Value	LUI	UUI
Afghanistan	14642.9	8054.7	22403.6	80.5	44.3	123.1	-3.4%	-38.5%	72.7%
Albania	1242.7	910.4	1671.5	96.1	70.4	129.2	25.7%	-9.4%	69.5%
Algeria	14044.3	10390.5	18610.9	62.6	46.3	83.0	24.4%	-15.4%	75.0%
American Samoa	41.1	29.9	55.7	149.5	108.8	202.9	26.3%	-14.4%	83.5%
Andorra	67.3	47.1	92.1	164.2	115.1	224.9	13.6%	-31.0%	77.2%
Angola	8617.3	5632.6	11830.0	63.7	41.6	87.4	13.0%	-31.9%	81.7%
Antigua and Barbuda	42.5	34.6	53.2	89.0	72.5	111.3	25.2%	-1.7%	57.3%
Argentina	38318.2	34748.7	42351.9	165.2	149.8	182.6	25.7%	11.7%	40.9%
Armenia	1688.9	1377.7	2025.6	114.3	93.2	137.1	-20.1%	-35.3%	-2.5%
Australia	14079.7	12594.1	15714.2	122.0	109.1	136.2	-11.8%	-21.5%	-1.1%
Austria	3004.4	2649.8	3394.4	74.5	65.7	84.1	-57.0%	-62.5%	-50.7%
Azerbaijan	8127.7	6419.8	10163.5	146.1	115.4	182.7	-10.1%	-29.6%	13.5%
Bahamas	403.2	314.9	513.4	196.9	153.8	250.8	42.8%	8.4%	85.8%
Bahrain	773.5	571.9	1001.4	83.7	61.9	108.3	36.2%	-3.8%	88.4%
Bangladesh	29420.3	18952.7	43585.7	34.1	22.0	50.6	7.9%	-35.6%	85.9%
Barbados	246.3	196.7	309.6	175.7	140.3	220.8	37.4%	7.7%	75.1%
Belarus	5727.2	4258.2	7729.7	131.4	97.7	177.4	-23.7%	-43.5%	2.4%
Belgium	4494.2	3960.3	5030.8	89.7	79.1	100.4	-27.4%	-36.6%	-17.4%
Belize	197.8	167.5	235.1	88.1	74.6	104.8	161.2%	112.5%	232.5%
Benin	2444.8	1656.9	3452.2	42.0	28.5	59.3	15.3%	-24.1%	69.8%
Bermuda	38.3	29.8	49.3	138.1	107.5	177.6	-8.2%	-31.7%	20.7%
Bhutan	185.8	92.6	285.7	42.7	21.3	65.6	35.5%	-21.6%	148.7%
Bolivia	4427.8	2911.8	6260.2	72.5	47.7	102.5	22.7%	-24.4%	90.3%
Bosnia and Herzegovina	2793.2	2151.9	3593.5	184.0	141.8	236.7	52.5%	13.5%	102.6%
Botswana	1568.2	914.3	2396.3	118.5	69.1	181.2	93.0%	7.2%	225.2%
Brazil	135880.6	128375.5	142679.1	117.7	111.2	123.5	46.8%	37.3%	55.9%
Brunei Darussalam	548.9	452.3	660.4	206.3	170.0	248.2	19.1%	-12.1%	55.7%
Bulgaria	7358.1	5565.1	9567.1	242.8	183.7	315.7	12.4%	-15.9%	49.2%
Burkina Faso	4347.1	3030.2	5927.0	42.5	29.6	57.9	27.7%	-13.9%	80.3%
Burundi	2797.8	1884.3	4084.5	51.0	34.4	74.5	-15.4%	-48.4%	48.7%
Cabo Verde	182.9	133.6	246.3	59.4	43.4	80.0	70.1%	22.5%	142.5%
Cambodia	10698.4	8105.1	14364.9	121.8	92.2	163.5	50.7%	-2.2%	144.0%
Cameroon	9041.2	5886.5	13200.5	62.9	40.9	91.8	14.5%	-29.8%	77.4%
Canada	18009.5	16063.4	20178.6	111.4	99.3	124.8	-1.7%	-13.3%	11.6%
Central African Republic	1313.7	877.9	1960.2	51.3	34.3	76.5	-5.7%	-38.2%	44.8%
Chad	2662.1	1865.8	3658.5	38.9	27.3	53.5	25.7%	-12.7%	84.8%
Chile	9026.8	7990.3	10129.7	98.2	86.9	110.2	39.8%	21.2%	60.4%
China	1304828.1	1099584.6	1526715.0	181.0	152.6	211.8	59.1%	27.3%	98.9%
Colombia	24888.1	18457.8	32920.0	99.9	74.1	132.2	46.6%	9.3%	93.4%
Comoros	255.3	145.7	366.9	68.3	39.0	98.2	38.1%	-29.7%	413.0%
Congo	2333.9	1450.2	3450.4	87.2	54.2	128.9	5.9%	-39.0%	91.6%
Cook Islands	6.1	3.6	8.4	74.9	44.2	103.2	14.3%	-36.3%	89.0%
Costa Rica	3482.8	2641.3	4566.3	139.6	105.8	183.0	116.1%	57.2%	187.4%
Croatia	2871.9	2138.7	3768.1	154.1	114.8	202.2	-3.0%	-29.4%	29.1%
Cuba	6651.3	5171.3	8283.7	125.3	97.4	156.1	25.5%	-2.5%	59.2%
Cyprus	490.8	404.5	589.1	71.2	58.7	85.4	21.6%	-7.0%	53.4%
Czechia	6507.0	5022.1	8286.8	135.5	104.6	172.6	-39.9%	-54.7%	-22.1%
Cote d'Ivoire	7388.9	4950.3	10234.5	56.6	37.9	78.4	9.3%	-31.4%	61.4%
Democratic People's Republic of Korea	23038.1	14564.4	35287.0	165.6	104.7	253.6	8.7%	-38.9%	86.2%
Democratic Republic of the Congo	16107.7	10269.0	24066.8	38.6	24.6	57.7	-5.1%	-44.1%	46.5%
Denmark	2607.0	2305.3	2923.8	101.7	89.9	114.1	-30.4%	-39.8%	-20.7%

Country	2019						Change of death rate		
	DALYs			DALY rate (per 100 000)			2019 versus 1990		
	Value	LUI	UUI	Value	LUI	UUI	Value	LUI	UUI
Djibouti	565.0	328.2	881.6	88.0	51.1	137.3	68.1%	-4.9%	184.4%
Dominica	38.2	29.1	49.8	112.4	85.6	146.7	45.2%	3.4%	99.3%
Dominican Republic	6935.7	4732.7	9616.9	120.2	82.0	166.7	107.4%	40.5%	191.8%
Ecuador	8665.7	6567.9	11356.7	94.3	71.4	123.5	86.6%	37.4%	145.1%
Egypt	38136.3	26799.8	52842.6	74.2	52.1	102.8	22.6%	-15.1%	71.1%
El Salvador	3284.0	2381.7	4440.6	100.1	72.6	135.3	110.2%	48.0%	186.1%
Equatorial Guinea	530.0	292.1	894.9	70.8	39.0	119.6	46.4%	-27.5%	169.6%
Eritrea	2718.3	1828.1	3963.3	78.7	52.9	114.7	51.3%	-6.3%	148.8%
Estonia	706.0	521.3	931.0	121.4	89.6	160.0	-17.7%	-40.9%	10.0%
Eswatini	592.3	336.4	916.6	97.8	55.5	151.3	81.0%	4.9%	194.0%
Ethiopia	26008.1	18947.9	35863.0	49.5	36.1	68.3	-26.3%	-52.3%	42.0%
Fiji	481.0	360.7	633.7	103.2	77.4	135.9	28.0%	-13.0%	90.9%
Finland	1690.1	1476.9	1926.5	72.4	63.3	82.5	-28.5%	-39.6%	-17.1%
France	28271.0	24833.6	31888.6	99.4	87.3	112.2	-13.9%	-25.2%	-2.0%
Gabon	908.4	581.7	1315.4	98.3	63.0	142.4	5.4%	-40.3%	79.9%
Gambia	378.2	250.3	544.5	33.9	22.5	48.9	37.2%	-20.6%	124.7%
Georgia	2858.1	2300.0	3494.0	172.8	139.1	211.2	6.7%	-17.2%	33.6%
Germany	38544.2	34663.8	42954.5	107.4	96.6	119.7	-21.5%	-30.3%	-11.9%
Ghana	11094.1	7631.8	15219.5	66.8	46.0	91.6	42.3%	-5.2%	117.8%
Greece	4486.9	4046.6	4950.8	100.4	90.5	110.7	35.8%	20.4%	51.8%
Greenland	57.4	44.2	76.2	215.8	166.1	286.6	-2.3%	-30.2%	41.0%
Grenada	71.1	57.8	86.5	131.2	106.6	159.8	40.1%	9.7%	76.3%
Guam	142.9	114.5	178.0	176.1	141.2	219.5	54.9%	14.8%	109.0%
Guatemala	8206.9	6305.8	10557.2	85.8	65.9	110.3	98.1%	48.0%	165.2%
Guinea	2406.5	1709.7	3274.5	42.1	29.9	57.2	19.1%	-21.5%	76.2%
Guinea-Bissau	574.4	423.1	775.2	60.7	44.7	81.9	3.7%	-31.9%	55.8%
Guyana	605.3	443.7	811.3	146.0	107.0	195.7	73.6%	23.3%	142.6%
Haiti	5218.1	3407.2	7574.4	79.6	52.0	115.6	-3.7%	-38.4%	44.4%
Honduras	2433.5	1442.4	3729.3	47.0	27.9	72.1	-0.1%	-41.2%	63.4%
Hungary	8849.8	6925.5	11154.1	199.3	156.0	251.2	-12.2%	-31.1%	11.5%
Iceland	106.1	90.5	123.3	65.0	55.4	75.5	-11.7%	-27.4%	6.1%
India	485009.4	404896.5	574274.0	63.9	53.3	75.6	36.5%	8.2%	65.3%
Indonesia	204752.7	146964.8	268911.1	143.4	102.9	188.3	66.1%	25.3%	123.8%
Iran	41486.0	38521.1	44988.9	87.8	81.5	95.2	64.4%	29.4%	105.5%
Iraq	17346.9	12509.5	24414.9	75.0	54.1	105.6	33.1%	-20.9%	121.9%
Ireland	2221.1	1908.0	2592.9	95.3	81.8	111.2	-25.1%	-37.6%	-11.8%
Israel	4102.5	3642.1	4616.2	93.7	83.2	105.5	-9.3%	-20.6%	3.8%
Italy	28751.0	26958.6	30462.3	113.4	106.3	120.1	-12.0%	-17.3%	-6.8%
Jamaica	2034.9	1506.5	2682.7	132.9	98.4	175.2	202.1%	118.8%	306.4%
Japan	69580.5	65208.5	72617.1	134.4	126.0	140.3	-19.3%	-23.5%	-16.0%
Jordan	5566.9	4519.2	6791.0	87.9	71.3	107.2	17.6%	-13.8%	55.3%
Kazakhstan	9592.0	7935.1	11377.6	103.3	85.5	122.6	-25.2%	-38.0%	-10.7%
Kenya	13645.4	10381.9	17492.1	52.4	39.9	67.2	79.4%	32.8%	140.3%
Kiribati	78.7	57.6	106.0	130.2	95.4	175.4	0.7%	-31.6%	49.8%
Kuwait	1622.3	1314.6	2004.9	55.7	45.2	68.9	32.7%	6.3%	68.3%
Kyrgyzstan	2133.6	1806.5	2497.8	63.5	53.8	74.4	-47.7%	-57.1%	-37.1%
Lao People's Democratic Republic	4850.6	3180.3	6736.8	124.5	81.6	172.9	19.4%	-23.4%	103.1%
Latvia	983.8	769.9	1265.3	120.1	94.0	154.5	-22.0%	-40.0%	0.6%
Lebanon	3341.6	2464.3	4387.1	125.1	92.2	164.2	30.7%	-18.6%	99.8%
Lesotho	961.5	605.6	1378.6	83.8	52.8	120.2	146.7%	33.8%	314.4%
Liberia	1016.3	606.7	1529.0	41.1	24.5	61.8	-3.1%	-42.0%	46.9%
Libya	5507.9	3849.5	7630.1	134.1	93.7	185.7	49.2%	-19.5%	156.5%

Country	2019						Change of death rate		
	DALYs			DALY rate (per 100 000)			2019 versus 1990		
	Value	LUI	UUI	Value	LUI	UUI	Value	LUI	UUI
Lithuania	1460.2	1120.5	1862.1	120.5	92.5	153.7	-15.8%	-35.4%	6.6%
Luxembourg	267.9	230.5	310.4	87.3	75.1	101.2	-41.3%	-51.1%	-30.5%
Madagascar	7475.2	5142.8	10336.4	56.6	38.9	78.3	-5.3%	-38.3%	40.5%
Malawi	3156.3	2082.6	4556.3	35.1	23.1	50.6	16.9%	-25.9%	84.4%
Malaysia	22003.6	15681.0	29308.5	126.0	89.8	167.9	28.4%	-11.4%	80.4%
Maldives	157.0	123.7	197.1	49.3	38.8	61.9	-9.9%	-42.6%	67.0%
Mali	4329.9	3005.5	5886.8	44.8	31.1	61.0	-0.7%	-31.6%	43.3%
Malta	168.3	141.5	197.1	85.4	71.8	100.0	7.2%	-12.3%	30.0%
Marshall Islands	43.2	28.8	59.8	142.6	95.1	197.5	54.7%	3.5%	117.7%
Mauritania	742.9	478.0	1085.0	38.3	24.7	56.0	-19.5%	-52.0%	37.1%
Mauritius	786.5	610.9	994.7	121.0	94.0	153.0	95.0%	48.1%	152.8%
Mexico	66064.6	56182.8	76696.7	99.7	84.8	115.7	101.4%	71.7%	135.5%
Micronesia	73.5	26.4	110.9	137.1	49.3	207.0	19.7%	-59.2%	87.6%
Monaco	24.9	18.3	34.6	175.0	128.7	242.5	4.5%	-29.1%	55.3%
Mongolia	1865.8	1376.0	2515.1	103.2	76.1	139.1	52.2%	7.1%	115.8%
Montenegro	336.9	267.7	412.3	113.9	90.5	139.3	6.5%	-19.3%	38.3%
Morocco	12098.5	8396.5	18505.5	63.1	43.8	96.5	47.2%	-6.6%	134.0%
Mozambique	6489.5	4121.0	9349.9	48.7	30.9	70.2	61.7%	2.6%	145.2%
Myanmar	39025.3	26238.3	54700.6	135.0	90.8	189.2	39.1%	-14.2%	114.1%
Namibia	666.8	411.9	1019.2	53.1	32.8	81.2	55.1%	-11.2%	177.2%
Nauru	10.4	5.9	14.3	184.8	105.6	254.7	-6.6%	-36.6%	35.5%
Nepal	5739.3	3741.4	8364.8	35.5	23.1	51.7	8.5%	-35.6%	73.6%
Netherlands	8751.7	7726.2	9871.6	116.6	102.9	131.5	-7.3%	-19.0%	5.2%
New Zealand	2692.2	2431.3	2986.3	135.6	122.5	150.4	-20.4%	-30.2%	-9.3%
Nicaragua	2221.3	1684.5	2814.4	63.4	48.1	80.3	38.1%	-2.0%	88.4%
Niger	2646.0	1746.3	3962.7	27.9	18.4	41.8	-15.3%	-43.9%	22.8%
Nigeria	36599.8	25172.7	51596.1	36.3	25.0	51.1	18.8%	-21.0%	84.8%
Niue	1.0	0.6	1.6	136.2	78.8	212.6	8.0%	-41.2%	81.7%
North Macedonia	1766.5	1355.6	2303.3	163.0	125.1	212.5	32.2%	-0.3%	75.4%
Northern Mariana Islands	37.5	26.9	50.6	182.9	131.4	246.9	6.0%	-35.3%	73.6%
Norway	2621.9	2381.3	2803.6	105.3	95.6	112.5	-22.2%	-29.3%	-16.5%
Oman	1272.1	939.5	1885.5	40.6	30.0	60.2	-1.2%	-43.0%	60.1%
Pakistan	85657.4	66731.7	108642.5	76.6	59.7	97.2	71.8%	25.1%	140.1%
Palau	12.4	8.6	16.7	133.4	92.2	180.1	37.9%	-15.3%	118.9%
Palestine	2822.0	2320.4	3361.5	111.3	91.5	132.5	16.1%	-23.1%	82.8%
Panama	1914.3	1384.0	2523.5	91.1	65.9	120.1	56.7%	10.6%	111.5%
Papua New Guinea	3688.0	2570.4	5138.8	72.5	50.6	101.1	26.1%	-10.3%	81.1%
Paraguay	3576.3	2574.5	4822.2	96.2	69.2	129.7	104.6%	40.0%	188.3%
Peru	13602.9	9765.4	18698.1	76.3	54.8	104.9	33.7%	-8.9%	93.9%
Philippines	100983.4	82568.9	124946.4	172.7	141.2	213.7	20.4%	-5.1%	53.0%
Poland	26932.7	22086.3	32028.5	148.0	121.4	176.0	-4.1%	-21.7%	13.6%
Portugal	7572.9	6645.9	8583.9	161.4	141.7	183.0	7.7%	-6.4%	24.4%
Puerto Rico	2378.5	1718.7	3215.1	150.1	108.4	202.9	37.2%	-2.3%	88.7%
Qatar	1019.4	743.2	1372.8	47.2	34.4	63.6	-3.2%	-39.5%	50.0%
Republic of Korea	27870.6	24461.3	31519.5	107.0	93.9	121.0	4.1%	-9.9%	20.8%
Republic of Moldova	2887.4	2412.6	3408.8	157.2	131.3	185.6	-18.6%	-32.7%	-2.6%
Romania	17075.1	13622.0	21488.2	197.2	157.3	248.2	41.3%	11.8%	76.4%
Russian Federation	109286.1	92474.4	127281.9	159.6	135.1	185.9	29.5%	11.0%	50.1%
Rwanda	3943.9	2635.6	5661.7	61.0	40.7	87.5	-9.0%	-47.4%	75.2%
Saint Kitts and Nevis	32.6	10.9	51.3	103.6	34.7	163.1	-11.7%	-70.1%	42.0%
Saint Lucia	104.7	85.2	127.8	111.9	91.0	136.5	51.9%	20.4%	88.0%
Saint Vincent and the Grenadines	75.8	62.9	91.2	132.6	109.9	159.5	80.9%	45.3%	119.9%

Country	2019						Change of death rate		
	DALYs			DALY rate (per 100 000)			2019 versus 1990		
	Value	LUI	UUI	Value	LUI	UUI	Value	LUI	UUI
Samoa	83.0	51.2	118.6	79.8	49.2	114.1	26.4%	-26.5%	108.7%
San Marino	18.0	10.8	29.0	118.7	71.3	191.7	20.7%	-31.5%	114.0%
Sao Tome and Principe	90.5	57.0	133.7	84.5	53.3	124.9	81.9%	7.2%	220.1%
Saudi Arabia	24229.2	17410.5	33729.2	99.5	71.5	138.5	115.5%	23.8%	273.3%
Senegal	3464.7	2240.9	4815.1	47.2	30.5	65.6	9.4%	-29.8%	63.4%
Serbia	7317.2	5546.6	9475.1	182.8	138.6	236.7	-1.4%	-29.4%	38.7%
Seychelles	134.9	112.2	161.7	249.0	207.1	298.5	96.5%	56.7%	147.5%
Sierra Leone	1648.0	1118.3	2285.9	39.7	26.9	55.0	13.9%	-25.8%	73.3%
Singapore	2487.8	2187.1	2814.0	80.9	71.1	91.5	-42.4%	-50.3%	-33.6%
Slovakia	4525.2	3301.9	6059.9	172.0	125.5	230.3	-1.9%	-28.8%	32.4%
Slovenia	1040.4	757.7	1392.4	116.1	84.6	155.4	-18.0%	-47.6%	23.2%
Solomon Islands	487.1	266.6	684.6	148.4	81.2	208.5	41.6%	-3.8%	100.4%
Somalia	3659.4	2170.7	6949.4	38.7	22.9	73.4	-11.5%	-47.5%	45.0%
South Africa	25405.8	21885.2	29486.5	83.4	71.9	96.8	-7.0%	-24.1%	15.8%
South Sudan	2751.8	1591.9	4583.3	65.3	37.8	108.7	13.6%	-29.8%	95.1%
Spain	25836.7	23036.6	29037.8	125.0	111.5	140.5	-0.8%	-13.3%	12.4%
Sri Lanka	5746.1	4103.9	7903.2	52.7	37.6	72.5	23.9%	-15.9%	78.3%
Sudan	11416.6	6740.6	17876.5	54.8	32.3	85.8	29.3%	-27.2%	115.3%
Suriname	426.4	335.5	535.8	147.9	116.4	185.9	66.1%	25.9%	133.6%
Sweden	4359.0	3970.4	4769.0	97.4	88.7	106.5	-15.1%	-24.2%	-6.0%
Switzerland	2977.6	2597.0	3354.4	74.6	65.0	84.0	-19.3%	-30.7%	-7.4%
Syrian Arab Republic	4341.9	3130.8	6022.0	58.5	42.2	81.1	1.1%	-37.3%	60.0%
Tajikistan	4835.0	3744.0	6160.2	97.3	75.3	124.0	-30.8%	-47.4%	-11.2%
Thailand	47154.6	33314.9	64019.9	132.1	93.4	179.4	34.5%	-6.2%	92.4%
Timor-Leste	563.2	179.9	816.1	86.3	27.6	125.0	38.8%	-65.0%	136.4%
Togo	1987.8	1268.1	2842.9	50.6	32.3	72.3	24.7%	-18.3%	80.6%
Tokelau	0.7	0.4	1.1	110.4	63.5	165.7	25.5%	-28.9%	100.5%
Tonga	25.4	17.6	35.7	51.9	36.1	73.1	34.0%	-12.2%	101.2%
Trinidad and Tobago	961.5	669.5	1319.7	138.4	96.4	190.0	28.4%	-10.8%	76.7%
Tunisia	4673.7	3245.8	6432.8	77.4	53.7	106.5	69.8%	8.0%	151.2%
Turkey	50299.1	39383.8	63594.5	110.2	86.3	139.3	-9.9%	-37.1%	29.1%
Turkmenistan	2164.0	1709.6	2814.1	82.1	64.9	106.8	-17.9%	-36.4%	7.6%
Tuvalu	7.0	4.6	9.9	115.8	75.9	163.9	7.4%	-30.5%	58.7%
Uganda	14139.2	10054.5	19583.7	74.9	53.3	103.7	105.6%	32.1%	227.3%
Ukraine	47078.5	38741.8	57603.0	226.5	186.4	277.2	-8.5%	-25.9%	13.7%
United Arab Emirates	7366.2	4925.0	10417.0	105.7	70.7	149.5	58.0%	-14.4%	185.3%
United Kingdom	34838.5	33720.3	36015.8	114.3	110.6	118.2	-12.8%	-16.2%	-9.7%
United Republic of Tanzania	17401.8	11794.3	25493.5	65.3	44.2	95.6	33.4%	-10.3%	94.6%
United States of America	205247.8	197213.9	213729.7	136.3	131.0	142.0	13.4%	8.4%	20.2%
United States Virgin Islands	89.9	64.7	127.0	207.0	149.0	292.4	26.1%	-14.0%	85.7%
Uruguay	2702.6	2407.7	3021.1	164.0	146.1	183.3	-1.6%	-15.6%	13.6%
Uzbekistan	16091.8	13383.7	19266.5	89.0	74.0	106.6	-6.8%	-23.2%	12.2%
Vanuatu	139.7	88.1	199.0	96.1	60.6	136.9	39.4%	-16.9%	123.6%
Venezuela	15599.3	11379.0	21016.5	108.4	79.1	146.0	68.8%	22.2%	128.7%
Viet Nam	80206.8	57338.9	109563.3	151.3	108.1	206.6	176.6%	73.0%	329.4%
Yemen	7883.3	5129.8	11271.7	50.4	32.8	72.1	27.7%	-28.7%	135.0%
Zambia	8532.4	5710.2	12084.6	94.5	63.3	133.9	35.9%	-14.6%	106.1%
Zimbabwe	7429.8	5020.0	10391.6	98.8	66.7	138.1	97.2%	27.1%	185.7%

DALYs, disability-adjusted life-years; 95% UI, 95% uncertainty intervals; LUI, lower uncertainty interval; UUI, upper uncertainty interval.
